# Automated strabismus evaluation: a critical review and meta-analysis

**DOI:** 10.3389/fneur.2025.1620568

**Published:** 2025-09-10

**Authors:** Emma M. Hartness, Fangfang Jiang, Gideon K. D. Zamba, Caroline Allen, Tara L. Bragg, Julie Nellis, Alina V. Dumitrescu, Randy H. Kardon

**Affiliations:** ^1^Carver College of Medicine, University of Iowa, Iowa City, IA, United States; ^2^Department of Biostatistics, University of Iowa, Iowa City, IA, United States; ^3^Iowa City VA Center for the Prevention and Treatment of Visual Loss, VA Health Care System, Iowa City, IA, United States; ^4^Department of Ophthalmology and Visual Sciences, University of Iowa, Iowa City, IA, United States

**Keywords:** strabismus, ocular misalignment, automated strabismus evaluation, meta-analysis, technological development, eye movement disorders

## Abstract

**Introduction:**

Adult strabismus has a wide range of etiologies and necessitates clinical evaluation for appropriate treatment. Advancements in eye tracking technology show promise for the development of clinically accurate, automated evaluation and diagnosis of peripheral and central causes of ocular misalignment. However, multiple barriers prevent the incorporation of automated devices into clinical use. This study aimed to perform a quantitative meta-analysis and qualitative assessment of published reports of devices capable of automated strabismus evaluation.

**Methods:**

A systematic search of the literature was conducted to identify reports of automated strabismus evaluation published between the years 1949–2025. Sixty-nine studies were identified through the literature search, and 17 of these studies qualified for statistical meta-analysis of automated device quality compared to gold standard clinical evaluation. We also analyzed factors affecting clinical use, including device portability, cost, and applicability toward patients with extreme angles of strabismus or anatomic variances, among others.

**Results:**

Meta-analysis demonstrated a pooled estimation of correlation of 0.87 [95% CI: (0.81, 0.91)] between results obtained by devices capable of automated strabismus evaluation in the literature and gold standard clinical evaluation. We identified advantages and limitations of previous models and offered guidelines to facilitate the advancement of device capabilities toward the level of gold standard expert clinical evaluation, and to facilitate the clinical implementation of these devices.

**Discussion:**

While barriers exist between experimental testing and clinical incorporation, automated strabismus technology shows promise for rapid, precise, and accurate evaluation of strabismus and has the potential to expand access to ophthalmic care in cases of low-resource or remote areas that lack local expert clinical personnel.

## 1 Introduction

Strabismus can be defined as a misalignment of the visual axes that may be congenital or acquired (1). The diagnosis has an estimated prevalence of 4% among the pediatric population and between 1 and 4% among the adult population worldwide (1–5). In individuals with normal ocular alignment, or orthophoria, both eyes can fixate on an object simultaneously. In those with strabismus, one eye is fixated on an object of interest, while the opposite eye is deviated away from the fixating eye (6). Strabismus may be either congenital or acquired in origin (3, 6). Congenital misalignment, which is often comitant (the misalignment is a constant, fixed amount in any direction of gaze), is the most common category of strabismus overall (3, 6). The etiology of congenital misalignment is incompletely understood but is suggested to arise from central nervous system pathways involved in processing and control of oculomotor function, including the lateral geniculate nucleus, midbrain fusion centers, striate cortex, and extrastriate cortical areas (3, 6). Acquired strabismus is typically incomitant and may be attributed to systemic conditions, such as vascular disease resulting in an aneurysm or ischemia, autoimmune disorders, demyelinating disease, systemic granulomatous disease, or muscular dystrophies (1–3, 6, 7). Misalignment can also arise as a sign of central nervous system infection, a neoplastic processes that raises intracranial pressure or affects the cranial nerves or extraocular muscles, or a cavernous sinus pathology (1–3, 6, 7). Additionally, acute trauma to the eye, extraocular muscles, or craniofacial structures can cause ocular misalignment (1, 3). Strabismus can result from adult-onset conditions affecting the tone, elasticity, or position of the extraocular muscles (e.g., thyroid eye disease, orbital inflammation, myositis, orbital or facial trauma, use of periocular implantable devices and age-related), or can re-emerge in adulthood as a decompensation of childhood strabismus, potentially with a history of surgery (1, 3, 6). Altogether, strabismus has a wide range of etiologies, and undiagnosed or new-onset strabismus warrants a timely and thorough evaluation to determine the cause and appropriate treatment for the best possible outcome (1, 3, 6).

Treatment of strabismus largely depends on the type and etiology of disease (1–3, 6, 7). Misalignment due to refractive error may be corrected with prescription lenses (1, 6). Often, strabismus requires surgical intervention to resolve diplopia or to improve a patient’s ability to make eye contact. The new onset of misalignment in older children and adults may suggest the need for further workup including imaging to assess for additional underlying pathology requiring interdisciplinary treatment (1, 6).

The gold standard measurement of ocular misalignment is achieved by using single and alternate cover tests and prisms, with alignment usually reported in prism diopters (8). Additional tests that assess for misalignment include the Hirschberg ratio or corneal light reflex, the Krimsky test, which uses prisms to center the corneal light reflex, the Brückner method, which uses an ophthalmoscope to assess for an asymmetric red reflex, the Hess screen test, the Lancaster red-green test, or synoptophore testing (7–10). Complete clinical evaluation of strabismus requires highly trained orthoptists, strabismus surgeons, or neuro-ophthalmologists who are trained in performing a sensorimotor exam and determining if additional systemic work-up or imaging is necessary (11, 12).

Multiple barriers exist between patients and appropriate evaluation of ocular misalignment. Evaluation of strabismus at any age should be timely, as undiagnosed strabismus can have consequences which range from decreased quality of life to significant morbidity or mortality, depending on the cause (4, 13, 14). Evaluation of strabismus in clinic is time-consuming and requires extensive clinical experience for examiners to accurately quantify misalignment (1, 11). Studies show that access to clinical experts trained in strabismus evaluation varies depending on geographical location and in some cases, socioeconomic status (15, 16). Additionally, the literature is lacking regarding guidelines for imaging in the setting of acute-onset misalignment, which often leads to unnecessary imaging and an inefficient use of healthcare resources (17).

Using automated systems for strabismus assessment would increase timely access for diagnosis and treatment for patients and reduce subjective measurement variability (12, 18). Concerning the pediatric population, automated strabismus and motility evaluation is under investigation as a tool to gain insight into infant eye movement, tracking, and cognitive development (19). Developing a clinically accurate, automated method of strabismus and ocular motility evaluation has become a popular field of technological research and development (8, 20–22). Previous devices that have been tested with the goal of assessing ocular deviation have applied a range of techniques, from the use of photographs to detect deviation in the nine cardinal gaze positions to the use of an automated application of the Hirschberg test, to the use of video-based pupil-tracking software to accurately estimate the degree of misalignment (21, 23, 24). More recently, the utilization of artificial intelligence and the adaptation of virtual reality head-mounted devices, many of which were originally developed for entertainment and gaming, have shown promise in the development of a portable, easy-to-use evaluation of ocular misalignment (25, 26). Despite these developments, multiple barriers prevent incorporation of these devices into clinical use, including the challenge of designing virtual reality headsets that fit both adults and children, the limitation of some devices that screen for the presence of strabismus without quantification or characterization of the misalignment, measurement of extreme degrees of deviation, evaluation of paralytic strabismus, accurate tracking of ocular structures in cases of ptotic eyelids or small eyelid fissures, rapid testing protocols, accurate automated software analysis of video recordings, cost of instrumentation, and portability of equipment (11, 22, 27, 28).

The objective of this study was to systematically review the literature for quantitative and qualitative evidence with which to evaluate the accuracy, reliability, portability, and feasibility of clinical implementation of devices that perform automated strabismus measurement. Primarily, we questioned how well automated strabismus devices perform quantitative measurement and characterization of strabismus compared to gold-standard clinical evaluation. Secondarily, we questioned what technical and contextual factors limit clinical implementation of technologies capable of automated strabismus evaluation. This review aims to identify advantages and limitations of previously proposed device models and to propose a framework for a device capable of automated strabismus measurement. This report also provides recommendations regarding the effective design of automated strabismus technology that compares to gold standard clinical evaluation. Additionally, this study proposes guidelines regarding the implementation of validation and feasibility studies to facilitate the incorporation of automated measurement technology into healthcare settings where clinical expertise on ocular misalignment is unavailable.

## 2 Methods

### 2.1 Literature search

**Figure 1:**
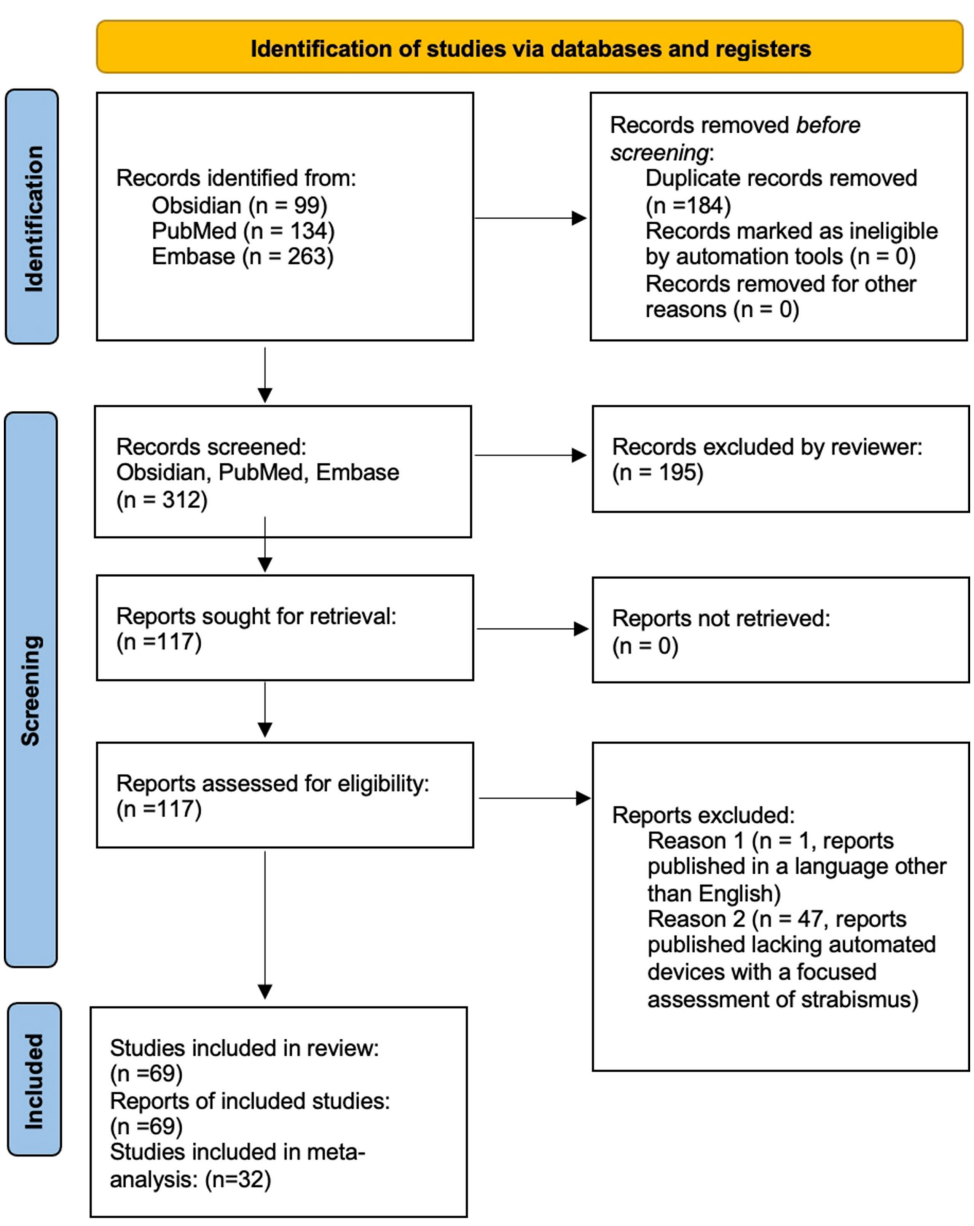
PRISMA diagram outlining literature review search methods.

A review was performed of reports published between the years 1949–2025 to analyze the available online published scientific literature describing devices capable of performing automated strabismus measurement that have been tested on either normal research participants, strabismus patients, or both. Considering the PICOS framework, this study examined how the assessment of strabismus by devices capable of automated strabismus evaluation in adult and pediatric populations compared to gold standard clinical evaluation. Outcomes considered included the accuracy and validity of strabismus detection and measurement in various gaze directions, as reported in various study designs that reflect the diversity of technological advancements reported in the literature. Data was collected through searches across the following platforms: Obsidian, PubMed, and Embase. Obsidian software was used to perform an advanced search. For this project, five folders were created in the Obsidian vault: Bibliographies, MeSH Terms, Original Bibliography, Project Notes, and Search Notes. After the initial literature search was conducted, individual notes were made for each MeSH Term assigned to the relevant articles. Then, individual notes were created for the citations of the articles and placed into the “Original Bibliography” folder. Each citation note included the article citation, bidirectional links to the notes of the assigned MeSH Terms, and the bibliography of the article. Notes were also created and linked for each citation on each bibliography list. These notes were placed in the Bibliographies folder. In the end, the MeSH Terms folder contained 157 notes, the Original Bibliography folder contained 52 notes, and the Bibliographies folder contained 814 notes. The most commonly used MeSH Terms were identified based on the number of links to the MeSH Term note, including “Humans,” “Child,” “Adult,” “Male,” “Female,” “Strabismus/diagnosis,” “Strabismus/physiopathology,” “Strabismus/diagnostic imaging,” “Reproducibility of Results,” “Vision, Binocular/physiology,” “Diagnostic techniques, ophthalmological,” “Fixation, Ocular/physiology,” “Esotropia/diagnosis,” “Exotropia/diagnosis,” “Vision Tests/methods,” “Image Processing, Computer-Assisted/methods,” “Sensitivity and Specificity,” “Vision Screening/instrumentation,” “Observer variation,” “Oculomotor muscles/pathology,” “Optics and photonics/instrumentation,” “Automation.” Two search statements were created based on these terms, the first as: (“Strabismus/diagnosis”[Mesh]) AND (((((“Diagnosis, Computer-Assisted”[Mesh]) OR (“Image Processing, Computer-Assisted”[Mesh])) OR (“Neural Networks, Computer”[Mesh])) OR (“Algorithms”[Mesh])) OR (“Pattern Recognition, Automated”[Mesh])), which produced 117 results, and the second as: (“Strabismus/diagnosis”[Mesh]) AND ((“Diagnosis, Computer-Assisted”[Mesh]) OR (“Image Processing, Computer-Assisted”[Mesh])), which produced 99 results. The date of last search using Obsidian was June 17, 2024. Additionally, the databases PubMed and Embase were used. PubMed search terms included “automated strabismus” and “automated strabismus evaluation.” yielding 134 search results. Embase search terms included ‘automated strabismus’ OR (automated AND (‘strabismus’/exp. OR strabismus)),” “‘automated strabismus evaluation’ OR “(automated AND (‘strabismus’/exp. OR strabismus) AND (‘evaluation’/exp. OR evaluation))” and “‘automated strabismus evaluation’ OR (automated AND (‘strabismus’/exp. OR strabismus) AND (‘evaluation’/exp. OR evaluation))” yielding 263 search results. After pertinent articles were extracted, their references were consulted for additional relevant literature. Duplicate search results were filtered from the included studies. Inclusion criteria included literature that was published and peer-reviewed, studies that were published in the English language, studies demonstrating the use of technology capable of automated strabismus detection and/or quantification, and studies validating or evaluating technology capable of automated strabismus detection and/or quantification. Exclusion criteria included studies that were unpublished or lacking peer review, studies that were published in a language other than English, or if the study pertained to automated assessment devices that evaluated conditions excluding strabismus. The studies were screened, read, and evaluated for inclusion in the study. At times, multiple studies from the same research group were included in the context of ongoing technological development by that group, or if distinct studies included updated hardware, software, or protocols of the same device, or included different technologies developed by the same research group, or included different subjects that were tested in the separate studies. The inclusion of these studies corresponded with our goal to analyze published studies pertaining to the development and clinical implementation of automated strabismus evaluation. The date of last formal search was October 30, 2024. Three reviewers screened records and assessed abstracts, and one reviewer assessed studies in full-length text for eligibility. In total, 69 articles met criteria for evaluation and were included in this review, and 32 studies were included (Figure 1). In the meta-analysis (see section “Statistical meta-analysis”). Studies not included in the meta-analysis were either included in the brief summary table (Table 1) or addressed in the Discussion.

**Table 1:** Summary of papers identified in literature search meeting inclusion criteria.

Study	Study population	Method	Eye tracking feature	Gaze position assessed	Fixation target distance	Validation method	Results	Limitations
Guyton et al. (44)	Adult and pediatric; Patients with strabismus; *n* = 6; Age range: 8–47 years	Remote infrared television camera	Pupil center	Nine cardinal gaze positions	80 centimeters	Hess-Lancaster; alternate cover test	Out of 1,188 horizontal and vertical measurements taken with all techniques, only 2.7% differed from the mean value for a particular test by more than 2.0 prism diopters, with the largest difference from the mean reported as 4.0 prism diopters.	True accuracy of remote haploscope testing and infrared television eye tracker testing is difficult to determine due to the lack of reported comparison with a manual, gold standard APCT.
Kault et al. (92)	Patient with strabismus; *n* = 1; Age unknown	Robinson’s Model, Reverse Model, and XEYE Package computer programs creating a model of strabismus and surgical planning.	Unspecified	Unspecified	Unspecified	Retrospective comparison of the computer model to the surgical planning and outcome of a prior case	“The case presented in Results should be regarded only as an illustration of XEYE since evaluation will require examination of a number of cases with strabismus measurements made under precisely standardized conditions.”	A limited number of patients (*n* = 1) limits the power of the study. A retrospective comparison makes conclusions of validity and accuracy difficult.
Campos et al. (93)	1 eye used as demonstration of the device model; Age unspecified	Remote infrared television camera	Pupil	Unspecified	Unspecified	Prior surgical planning and outcome	Descriptive study	Descriptive study
Thompson et al. (94)	Adult and pediatric; Strabismus patients; *n* = 38; Age range 6–81 years	Automated Hess screen test, remote device (AHS)	Patient-operated button to signal viewing target is centered on screen	Nine cardinal gaze positions	25 centimeters	Electronic Hess screen test (EHS)	The electronic Hess screen test results were found to be slightly more accurate than the automated Hess screen test in the production of an accurate strabismus diagnosis.	“[T]he panel of experts were all experienced at interpreting the motor fields measured using an EHS but had no previous experience interpreting AHS data.”
Schiavi and Orciuolo (95)	Age, Strabismus status, and number of patients unspecified	Remote infrared television camera	“Position of pupil relative to second image”	Primary	Distance (5 meters) and near (33 centimeters)	Validation method not discussed; Largely descriptive paper of new automated strabismus measurement model	No statistically analyzed quantitative results available.	Descriptive paper lacking objective numeric measurements of tested patients for comparison to gold standard APCT.
Miller et al. (96)	Adult; Non-strabismus participants with induced/simulated strabismus; *n* = 10; Age unspecified	Remote infrared video camera	Corneal light reflex compared to pupillary axis/ Hirschberg test	Primary	1 meter	Known value of artificially-induced strabismus	“Statistically significant linear correlation of Hirschberg horizontal reflex deviation with asymmetric fixation of pseudo-esotropia…(*p* < 0.05).”	(Discussed by the authors:) A spherical cornea was assumed to calculate centroid of Purkinje reflex; Most pupils appeared ellipse in shape, causing potential decentration of the image plane with respect to the eye midpoint on imaging;
Bos and de Graaf (43)	Adult; Strabismus status unknown; *n* = 7; Age range 19–45 years	Head-mounted goggles with video oculography	Details within iris structure	Primary	2 centimeters	Control “reference image”	“The implementation, applying averaging over ocular torsion determined in partitioned iris images, yields a theoretical resolution of 5′ of arc. In a control experiment with an artificial eye, the accuracy showed to be better than 14′ of arc.”	Limited sample size and without testing on patients with torsional strabismus.
Miller et al. (49)	Adult; Non-strabismus participants with induced/artificial strabismus; *n* = 18; Age range 20–40 years	Remote camera employing the Bruckner test	Coaxial fundus reflex	Primary	254 centimeters	Known value of artificially-induced strabismus	Results shown only in graph format (see original paper), numerical data not provided in table or manuscript.	Pupillary size assumed constant, but observed to change image to image; Unexplained variation of pupil brightness during testing; Authors noted significant camera noise.
Hasebe et al. (34)	Adult and pediatric; Strabismus patients; *n* = 87; Age range 4 months-75 years	Remote infrared television camera	Corneal light reflex, pupillary center (decentration of light reflex from pupil center measured)	Primary	140 centimeters	APCT	“The 95% limits of agreement between the Hirschberg measures and the PACT were within ±7.8° or ±13.7 PD. The average (± SD) Hirschberg ratio was 12.3 ± 1.2°/mm or 21.8 ± 2.1 PD/mm.”	Measurement was limited in participants with large angles of deviation; Reported measurement error due to intersubject variance in Hirschberg ratio.
Hunter et al. (97)	Adult; Non-strabismus participants; *n* = 16; Age unspecified	Remote reitnal birefringence scanning	Retinal birefringence	Nine cardinal gaze positions	1.5 meters	Normal subjects tested, some but not all (unspecified), had examination by ophthalmologist confirming normality	“RBS can be used for remote, noninvasive, continuous monitoring of true foveal fixation within 61°, without strict restrictions on head position or the need for head-mounted appliances.”	“Although many of the subjects had documentation of normal eye examinations including normal ophthalmoscopy, this was not performed routinely on all subjects.” The study lacks comparison with gold-standard measurement for validation.
Simons et al. (45)	Pediatric; Unknown strabismus status; *n* = 100; Age range 4 months to 12 years	Static photograph	Corneal light reflex	Primary	1 meter	APCT	The range of sensitivity of the MTI Photoscreener (screen for amblyogenic risk factors) in detecting strabismus was: 23 to 50%. Among clinical observers, the range of sensitivity for detecting strabismus was: sensitivity = 23 to 50%.	“The photoscreener had a low sensitivity even for manifest strabismus.” The authors also note the importance of adequate pupil size for effective screening, which could limit testing on patients with abnormal irises.
Scott et al. (98)	Monkey; *n* = 1	Head-mounted liquid crystal shutters that perform automatic occlusion of eyes for alternating cover test	Ocular misalignment during cover tests	Primary	50 centimeters	Alternating cover test, single cover test	“The shutters produced occlusion of each eye as effective as that of an opaque plastic occluder used in previous experiments that required monocular viewing. Heterotropias were detected and recorded in monkeys and closely resembled those observed in human patients. It was also possible to detect heterophorias by actuating the shutters alternately.”	Small sample size, not tested on humans with strabismus, strabismus assessed only in primary gaze.
Schaeffel (66)	Adult; Non-strabismus participants; *n* = 24; Age range 25–47 years	Remote infrared video camera	Corneal light reflex	Multiple gaze positions to calibrate kappa angle and Hirschberg ratio	90 centimeters	No gold-standard testing; Data compared to data in prior literature	Angle kappa and Hirshberg ratio were highly correlated in both eyes of the subjects.	The authors note that the gaze tracker requires a smooth cornea and cannot be used in patients with a history of refractive surgery. Data was not compared to gold-standard measurement, limiting validity.
Hunter et al. (40)	Adult; Non strabismus patients (*n* = 21), Strabismus patients (*n* = 4), Age range 20–58 years	Remote retinal birefringence scanning	Retinal birefringence	Primary	45 centimeters	Previous diagnosis strabismus	Detected binocularity (representing alignment) by retinal birefringence was significantly reduced in participants with strabismus compared to non-strabismus participants, suggesting the device may function as a screening tool.	Lower scores of binocularity were found in participants with smaller pupils, likely (per the authors) as a result of reduced light detection versus misalignment. Also, signal quality was noted to be lower in those with uncorrected myopia.
Hunter et al. (35)	Adult, Non-strabismus participant (*n* = 1), Strabismus patient (*n* = 1)	Remote retinal birefringence scanning	Retinal birefringence	Four ordinal directions	40 centimeters	Data from strabismus patients compared to data collected from non-strabismus patients within the same study	Measured binocularity was significantly reduced in participants with strabismus compared to non-strabismus participants, supporting the use of the device as a screening tool.	Lack of quantitative numerical data provided in the study. Lack of comparison between the study results from patients with strabismus with gold standard clinical testing.
Nassif et al. (50)	Adult; Non-strabismus participants (*n* = 40); Strabismus patients (*n* = 20); Age range 22–79 years	Remote retinal birefringence scanning	Retinal birefringence	Primary and the four ordinal gaze directions	40 centimeters	Reported “gold standard” clinical evaluation by orthoptist	“Good binocular alignment was appropriately detected in all control adults, while all adults with constant strabismus received a “refer” score.”	The near point of fixation utilized by the screening device necessitated accommodation, which may be limited in older adults.
Vaswani and Mudgil (99)	Non-strabismus participants; *n* = 70; Mean age: 16 years	The test was developed with binoculars with prisms and colored filters used to subjectively orient parallel lines on remote computer screen.	Subjective report of research participant/ orientation of parallel lines	Primary	2 meters	Unspecified	“The mean degree of cyclodeviation tilt in the right eye was 0.6 degrees for monocular viewing conditions and 0.7 degrees for binocular viewing conditions, with a standard deviation of approximately one degree. There was no statistically significant difference between monocular and binocular viewing,” supporting that the test may be used in the detection of cyclovertical strabismus.	Authors reported difficulty in testing outside of primary gaze position (which would present.difficulty in cases of patients with compensatory head postures). The test also cannot be used to test patients with constant diplopia due to their lack of fusion in primary position.
Van Eenwyk et al. (100)	Adult and pediatric; Unknown strabismus status; *n* = 610, Age range 6 months to 6 years	A remote video-based system combining Brückner pupil red reflex imaging and eccentric photorefraction that captured images analyzed by artificial intelligence	Brückner reflex	Primary	Unspecified	“Strabismus examination” by a clinician	For a ‘refer/do not refer” result, the system showed an accuracy of 77%, compared to the “gold standard” clinical examination and correctly identified 82% of strabismic individuals,	Suggetss that the device can be used to screen for, but not quantify or characterize, strabismus.
Han et al. (71)	Adult; Non-strabismus participants; *n* = 15; Age range 19–65	Remote infrared video recording system	Limbus	Primary	40 centimeters (Maddox rod), 45 centimeters (limbus tracking system)	APCT, Maddox rod test	“Responses objectively recorded using the limbus tracking system exhibited similar standard deviations to the Maddox rod and the alternate cover test techniques.”	The authors note that the use of a target for binocular fixation may affect the accurate assessment of phoria in their study (see study for full details).
Model et al. (101)	Adult and pediatric; Non-strabismus participants; *n* = 48; Age range:18–57	Remote two-camera video tracking system that performs a fixation-free measurement of the Hirschberg ratio	Pupil center and three corneal reflexes	None- fixation-free procedure	85 centimeters	Fixation-free measurements compared to fixation-based Hirschberg measurements	Fixation-free and fixation-based protocols were highly correlated (*r* = 0.95; *p* < 0.001) showed repeatability and consistency.	Study does not examine strabismus according to APCT gold standard measurements, however, is an important study for fixation-free testing of alignment in infants.
Model and Eizenman (46)	Pediatric; Non-strabismus participants; Age rang 6–16 months	Remote infrared video cameras used to measure Hirschberg ratio and angle kappa	Corneal light reflex	Primary	85 centimeters	This study measured for repeatability between measurements of the Hirschberg ratio and angle kappa	“The average difference between two independent measurements of eye misalignment was −0.27° ± 0.38° and the 95% limits of agreement for repeated measurements were ±0.75. “The AHT procedure can provide more accurate measurements of ocular misalignment than the standard HT. It may, therefore, enable early and reliable detection of infantile esotropia that may lead to early treatment and increase the chances for normal visual development in these patients.”	Lack of testing on patients with strabismus and comparison to gold standard APCT.
Almeida et al. (72)	Ages unspecified; Non-strabismus participants (n-30); Strabismus patients (*n* = 15)	Static photograph	Light reflection on cornea	Primary	40–50 centimeters	“cover test”	“The methodology has produced results on the range of 100% sensibility, 91.3% specificity and 94% for the correct identification of strabismus. on digital images obtained from the Hirschberg test.”	Patients were excluded from testing with the following conditions, (among others listed in the study): horizontal or vertical yaw above 15 degrees, cornea or limbus aberrations, presence of nystagmus, inability to achieve 40s of arc on Titmus stereoscopic visual acuity test, inability to achieve 1.0/1.0 on the Snellen table.
Yang et al. (78)	Adult and pediatric; Strabismus patients; n-32; Age range 0.5–58 years	Static photograph	Corneal light reflex and limbus	Primary	0.33 meter	“2 independent ophthalmologists measured participants with Krimsky test and prism and alternate cover test”	“The 95% limit of agreement of inter-observer variability was 63.58 (6.1 prism diopters (PD)), 63.18 (5.4 PD) and 61.58 (2.6 PD) for the Krimsky test, PCT and the 3D Strabismus Photo Analyzer, respectively. The test–retest reliability was 62.88 (4.9 PD) for the 3D Strabismus Photo Analyzer versus the Krimsky test. The results of the Krimsky test and 3D Strabismus Photo Analyzer showed a strong positive correlation.”	Analysis limited to assessment of manifest strabismus in primary gaze, and the authors discuss that the software may not be able to measure more extreme deviations of gaze.
Awadein (102)	Adult; Strabismus patients; *n* = 82; Age range 22–56 years	Computerized version of the Lancaster red-green test	Patient subjective response	Nine cardinal gaze positions	40 centimeters and 100 centimeters	Standard Lancaster red-green test	“The measured vertical and torsional deviation in the conventional test showed good agreement with both versions of the computerized test (limits of agreement < 5Δ for vertical measurements and < 3° for torsional measurements).”	APCT is considered the gold standard for strabismus assessment.
Yang et al. (60)	Non-strabismus participants (*n* = 30); Strabismus patients (*n* = 60); Ages unspecified	Remote infrared video camera with selective wavelength filter measuring angle kappa	Limbus, pupil, corneal light reflex	Primary	0.33 meter	APCT	“Results of the PCT and selective wavelength filter analysis showed a strong positive correlation (R = 0.900, *p* < 0.001).”	Authors discuss that the software may not be able to assess patients with extreme degrees of misalignment, nystagmus, torsion, or abnormalities of ophthalmic anatomy.
Khumdat et al. (103)	Pediatric; Unknown strabismus status; Age range 11–17 years	Static photograph	Corneal light reflex and limbus	Primary	1 meter and 6 meters	Examination by “three specialists,” unspecified.	The methodology has produced results on the range 94.17% of accuracy, 97.23% of sensitivity and 73.08% of specificity.	The assessment may be limited by occlusion of the eye surface by eyelids, hair, inadequate illumination or insufficient contrast.
Silbert and Matta (36)	Pediatric; Unknown strabismus status; *n* = 151; Age range 1–6 years	Infrared static photograph	Measuring noncycloplegic refraction in partially dark-adapted mid-dilated pupils	Primary	1 meter	“patients found to have amblyopia or amblyopia risk factors based on the 2003 AAPOS referral criteria” (including strabismus)	“A total of 151 children were included. The Spot had a sensitivity of 80% and specificity of 74%. With the revised 2013 AAPOS referral criteria, the sensitivity was 87% and specificity was 74%.”	“This study was limited by the ophthalmology clinic setting and the screening of an enriched population.” (see paper)
Priglinger et al. (104)	Pediatric; Strabismus patient, *n* = 1 real patient, 3 simulated patients; Age: 1 patient followed from age 6 months to 7 years	Patient results were compared to computer-generated simulated patients	Not discussed	Nine cardinal gaze positions	Unspecified	Prism cover test, Hess-Lancaster test	Quantitative values were provided in Figures (see paper); The device software was used to proposed a diagnosis for underlying strabismus.	Limited number of patients tested limits the power of the study.
Irsch et al. (105)	Adult and pediatric; Non-strabismus participants (*n* = 2), Strabismus patients (2); Age range 3–67 years	Remote retinal birefringence scanning	Retinal birefringence	Primary	Unspecified	Results (identification of strabismus during screening) compared to a previous diagnosis of strabismus	“Feasibility tests of focus detection with our new PVS using the improved target system with accommodation control suggest that the device has the potential to detect spherical focus within +/− 1.00 D”	Device demonstrates ability to screen for strabismus, but lacks ability to quantify or characterize strabismus.
Jost et al. (37)	Pediatric; Unknown strabismus status; *n* = 300; Age range 2–6 years	Remote retinal birefringence scanning	Retinal birefringence	Primary	35–40 centimeters	“Gold standard” cover testing	“The sensitivity of the PVS to detect strabismus and amblyopia (0.97; 95% CI, 0.94–1.00) was significantly higher than that of the SureSight Autorefractor (0.74; 95% CI, 0.66–0.83). Specificity of the PVS for strabismus and amblyopia (0.87; 95% CI, 0.80–0.95) was significantly higher than that of the SureSight Autorefractor (0.62; 95% CI, 0.50–0.73).”	“[The] present study was conducted in a clinical setting, with a cohort enriched in children affected by the targeted conditions of strabismus and amblyopia; therefore, this study cannot directly assess the performance of the PVS in a primary care screening setting”
Lim et al. (32)	Strabismus patients; *n* = 120; Age unspecified	Static photograph	Corneal limbus	Nine cardinal gaze positions	6 meters	Two independent observers measured the degree of inferior oblique muscle overactivation via a specified clinical grading scale, and interobserver reliability was measured	“The 95% limit of agreement of interobserver variability for the degree of inferior oblique muscle overaction was ±1.76 degrees, and ICC was 0.98. The angle of inferior oblique muscle overaction showed significant correlation with the clinical grading scale (R = 0.549, *p* < 0.001).”	Authors discuss that measurement error may arise due to clinician variability, that this method calculated inferior oblique overactivation using a 2D model, while the eye is 3D, that nine cardinal gaze position photographs may be difficult to obtain in patients with small eyelid fissures or in small children who are less cooperative with testing.
Garry and Donahue (106)	Pediatric; Unknown strabismus status; *n* = 155, Age range 2–9 years	Infrared static photograph	“The output from the screening instrument consists of 7 output values for each eye, including estimates of spherical and cylindrical refractive error, axis, and gaze vector.”	Primary	3 feet	“Participants.received a gold standard pediatric ophthalmic examination, consisting of an assessment of.strabismus”	“Spot was 89% sensitive and 71% specific in detecting amblyopia risk factors.”	“One limitation to our design involved using an ophthalmology clinic patient population as opposed to a more typical screening population in the field with high numbers of normal children.”
Peterseim et al. (107)	Pediatric; Unknown strabismus status; *n* = 444; Age range 1–16 years old	Infrared static photograph	Unspecified	Primary	3 feet	“A comprehensive examination was then performed, including.stereopsis and motility evaluation.”	“Compared to the ophthalmologist’s examination, the Spot sensitivity was 87.7% and the specificity was 75.9% in detecting amblyopia risk factors.”	“This study is limited by our testing of a high-risk population, which would be expected to decrease testability and alter the positive predictive value and negative predictive value.”
Seo et al. (62)	Unspecified	“Infrared camera and liquid crystal shutter glasses to simulate cover test and the digital video camera to detect the deviation of the eye.”	Pupil	Primary	Unspecified	Unspecified	This study is largely descriptive; Objective numerical data not provided.	Results given not compared to gold standard cover-uncover test. Number of tested patients not specified apart from one patient photographed.
Almeida et al. (20)	Strabismus patients; *n* = 40; Age range unspecified	Static photograph	Limbus vs. corneal light reflex	Primary, four ordinal gaze directions	Near: 40–50 centimeters, Distance: 6 meters	“The method’s accuracy was evaluated by comparing to the diagnoses presented by the specialist.”	“[The] method was demonstrated to be 88% accurate in esotropias identification (ET), 100% for exotropias (XT), 80.33% for hypertropias (HT), and 83.33% for hypotropias (HoT). The overall average error was 5.6Δ and 3.83Δ for horizontal and vertical deviations, respectively, against the measures presented by the specialist.	The Hirschberg test (upon which the automated test is based) is limited to patients who present with tropias.
Peterseim et al. (108)	Adult and Pediatric; Unknown strabismus status; *n* = 444; Age range 11 months-19 years	Remote video camera	Gaze criteria based on degrees displacement from the pupil center to the corneal light reflex.	Four ordinal gaze directions	3 feet	“A comprehensive examination was then performed, including.stereopsis and motility evaluation.[by] four experienced pediatric ophthalmologists.”	“The sensitivity of the Spot to detect AAPOS-threshold strabismus was 77.17%; the specificity, 93.73%.”	“children with intermittent strabismus may have had straight eyes [during screening] but later appeared to have constant strabismus to the ophthalmologist on examination. In our study, these children would be included as false negatives. Conversely, children identified as having strabismus by the Spot may have demonstrated abnormality in “gaze” during the screening but are not included as AAPOS-threshold strabismus positive because their strabismus was intermittent on examination. These children with intermittent strabismus would therefore be considered false positives.”
Otero-Millan et al. (77)	Strabismus (torsion) patient; *n* = 1; Age unspecified	Head-mounted infrared video camera	Pupil center	Primary, +/− horizontal degrees, +/− vertical degrees, 35 degrees horizontally, 50 degrees horizontally	Unspecified	Scleral annulus search coil and video oculography	“The current setup operates binocularly at 100 Hz with noise <0.10 degrees and is accurate within 20 degrees of gaze to the left, to the right, and up and 10 degrees of gaze down.”	“First, at some gaze positions… the eyes might be largely occluded by the eyelids and eyelashes. Second, during fast movements of the head, any head-mounted display may suffer from slippage of the goggles on the head… Other limitations are… when changes in pupil size shift the relative position of the centers of the pupil and the iris, when pupils have very nonelliptical shapes, and when pupils are very small and the corneal reflection from the light-emitting diode illumination completely covers them… though with more than 20 subjects we have yet to find a subject in whom these issues precluded reliable measures of torsion.”
Kumar et al. (109)	Adult; Non-strabismus participants (*n* = 8); Strabismus patients (*n* = 8); Age range 50–70 years	Remote infrared video camera	Pupil center	Primary plus five unspecified positions in horizontal and vertical positions	50 centimeters	Previous diagnosis of gaze palsy or abnormality, unspecified	“Our preliminary feasibility study with eight pairs of chronic (months) stroke survivors and healthy individuals revealed that gaze related indices in response to both static and dynamic visual stimuli may serve as potential quantitative biomarkers for stroke assessment.”	“Our preliminary feasibility study had some limitations. The gaze-related indices used in this study, though showed variations between the two groups of participants, did not have statistical significance, since our present study lacked sample power and our data was quite diffused. Also, we could have access to detailed neuroimaging reports of only two stroke patients which restricted us from doing in depth analysis of mapping one’s gaze-related indices to localized lesions in the brain. “
Valente et al. (52)	Strabismus patients; *n* = 7; Age unspecified	Remote video camera	Pupil	Primary	50 centimeters	“Specialist diagnosis,” unspecified	“To detect the presence of strabismus, the proposed method achieved a specificity value of 100%, and (2) a sensitivity value of 80%, with 93.33% accuracy in diagnosis of patients with exotropia. This procedure was recognized to diagnose strabismus with an accuracy value of 87%, while acknowledging measures lower than 1Δ, and an average error in the deviation measure of 2.57Δ”	“In detection of eye region and pupil location stage… the methodology failed in six cases due to situations in which the color of the pupil region, in dark eyes, was similar to the color above the pupil region. Another reason was that the shadow influence due to the close proximity of the patient’s body parts relative to the eye position in relation to the light source in the room.”
Maor et al. (42)	Pediatric; Strabismus patients (*n* = 409); Age range 3–9 years	Static photograph	Corneal light reflex	Primary	40–70 centimeters	“All children underwent orthoptic assessment including.stereoacuity, cover/uncover and alternate prism cover test, and eye movement assessment.”	“In children with phorias, the mean corneal light reflection location difference between the eyes was −0.10 ± 0.14 mm in primary position and −2.02 ± 0.39 mm in off-center fixation. Using a threshold of ±0.5 mm on either side of zero for central and of 2 mm for off-center fixation, sensitivity to detect strabismus increased from 65.6% in central to 79.3% in off-center fixation, respectively. The calculation of specificity will require inclusion of a population of individuals without strabismus.”	“Selection bias was introduced by choosing 34 cases out of a consecutive series of 52 cases and selecting images that allowed easy manual image processing using generic image analysis software. Cases of dark irides (poor contrast between pupil margin and pupil), poor eyelid opening, and poor target fixation were eliminated because it was not possible to obtain accurate measurements from these photographs. Another type of selection bias was the inclusion of predominantly esotropic participants… Detailed analysis and correlation of orthoptic measurements of strabismus and camera-based measurements were not meaningful because the algorithm aims to detect manifest strabismus, whereas the orthoptic alternate prism cover test measurements taken reflected the sum of manifest and latent strabismus.”
Nesaratnam et al. (54)	Adult; Strabismus patients; *n* = 3; Age range 22–73 years	Head-mounted virtual reality headset	Lees screen test: foveal position	Primary	Unspecified	Lees Screen test (modification of the Hess screen test)	“The pattern of deviation obtained using the virtual reality-based test showed agreement with that obtained from the Lees screen for patients with a fourth nerve palsy, comitant esotropia, and restrictive thyroid eye disease.”	Limited number of patients tested limits the power of the study.
Kim et al. (33)	Adult and pediatric; Non-strabismus participants (*n* = 30), Strabismus patients (*n* = 30); Age range 5–74 years	Computerized torsion test administered on liquid crystal display remote monitor; participants wore red–green filter spectacles (red–green glasses used for the Worth-four-dot test or LRGT)	Subjective reporting of visual images by subject	Primary: torsion testing	50 centimeters	Lancaster red green test, double Maddox rod test	“Both the DMRT and CTT showed no significant test–retest differences in the strabismus and control groups. The DMRT and CTT results demonstrated an acceptable agreement. The reliability of the CTT was better than that of the DMRT. The LRGT showed low sensitivity for the detection of ocular torsion compared with the DMRT (40.0%) and CTT (39.1%).”	“[T]he patients with strabismus had various disorders, which could have resulted in variable test outcomes. Second, the age range of participants was wide… which may have affected the reliability of results in very young and aged patients.”
Weber et al. (38)	Adult and pediatric; Non-strabismus participants (*n* = 17), Strabismus patients (*n* = 41); Age range 6–81 years	Head-mounted, infrared video goggles	Pupil	Nine cardinal gaze positions	0.5 meter	Hess screen test	“There was good agreement between the strabismus video goggles and the Hess screen test in the measurements of horizontal and vertical deviation (intraclass correlation coefficient horizontal 0.83, 95% confidence interval [0.77, 0.88], vertical 0.76, 95% confidence interval [0.68, 0.82]). Both methods reproduced the characteristic strabismus patterns in the 9-point grid. In contrast to Hess screen testing, strabismus video goggle measurements were even possible in patients with comitant strabismus and visual suppression.”	“[The] goggles… have been designed for an average-size head [and]… they did not fit all patients equally well… The current goggles prototype is not equipped to correct for refractive errors… sometimes a shadow image of the laser target was perceived by the occluded eye… Currently, the software analyzes only horizontal and vertical deviations, and ocular cyclotorsion has not been implemented yet.”
Chen et al. (110)	Adult and pediatric; Non-strabismus participants (*n* = 15), Strabismus patients (*n* = 10); Age range 3–63 years	Remote video camera	Unspecified	Nine cardinal gaze positions	50 centimeters	“Ophthalmologist’s diagnosis”	“Experimental results on the dataset demonstrate the effectiveness of the proposed system for strabismus diagnosis.” (See paper for details)	“The limitation of our system is that it cannot yet precisely measure the strabismus angle as the cover test with a prism, though the relative severity is quite accurate.”
Chen et al. (111)	Adult; Non-strabismus participants (*n* = 25), Strabismus patients (n = 17); Age range 25–63 years	Remote video camera, convolutional neural networks	Unspecified	Nine cardinal gaze positions	50 centimeters	“[The participants] have been diagnosed by a professional ophthalmologist, and the diagnosis results are used as ground truth in this paper.”	“Experimental results demonstrate that… strabismus can be effectively recognized by our proposed method.” (see paper for details)	Objective numerical data not explicitly provided in comparison to gold-standard APCT results.
Chopra et al. (57)	Adult; Non-strabismus participants (*n* = 15), Strabismus patients (*n* = 15); Age range 33–67 years	“[Binocular OCT:] [T]he corneal vertex reflection in the fixing eye. was used as a surrogate for the visual axis. A line was drawn between the pupil margins at the posterior epithelium of the iris for both eyes. The angle between the lines was calculated as the angle of deviation”	Corneal vertex reflection	Primary	Unspecified	APCT	“The APCT and OCT measurements were strongly correlated for the horizontal (Pearson *r* = 0.85; 95% CI, 0.60–0.95; *p* < 0.001) and vertical (*r* = 0.89; 95% CI, 0.69–0.96; *p* < 0.001) deviations.”	“Inability to ascertain heterophoria or heterotropia… all participants with strabismus had a constant deviation. Those with intermittent deviations may not be identified using the current prototype setup… Refractive error can affect the size of the deviation, and the inability to correct cylindrical error may contribute to the differences that were observed between the methods”
Thorisdottir et al. (112)	Adult; Unknown strabismus status; *n* = 19; Age range 27–78 years	Digital KM screen test: The patient wears red-green goggles and performs the test on a remote computer screen	“The positions indicated by the patients are recorded on the computer.”	“Twenty-five different points are used to test each eye, creating a primary position as well as an inner and an outer quadrant at 15° and 30° from the primary position.”	1 meter	Hess and Lees screen tests	“No significant differences were found between the results obtained by all three tests [*n* = 19 (*p* > 0.05)].”	“There are several limitations… Examiners [were not] masked to the results [of all three tests]… Second, our study cohort was small, and only half the group repeated the tests for testing the level of difficulty and duration but not for repeatability of the test results. Third, with congenital deviations, a habitual head posture can be difficult to overcome during testing.”
Mestre et al. (61)	Adult; Strabismus patients; *n* = 30; Age range 23–33 years	Remote infrared video eye tracker	Corneal light reflex	Primary	40 centimeters	Cover-uncover test and modified Thorington test	“The signed mean differences between the heterophoria measured with the three tested methods were considerably close to 0 PD, which means that on average none of the methods were clearly biased toward more esophoria or exophoric values.” (See paper for more details)	The paper discusses an inability to detect and/or measure properly paralytic heterotropias, since in these conditions the secondary deviation (the deviation when the paretic eye is fixating) is always greater than the primary deviation.
Yoo et al. (75)	Pediatric; Non-strabismus participants (*n* = 75), Strabismus patients (*n* = 83); Age range 0–4 years	Static infrared photograph	Corneal light reflex	Primary	Near: 0.33 meter, Distance: 5 meters	APCT	“The testability of infrared photographs using selective wavelength filters in children under 4 years of age was 85.6%. The mean angle of esodeviation was 11.3 ± 4.0 PD by manual measurements and 11.5 ± 4.4 PD by the infrared photograph analysis. Manual measurements and the infrared photograph analysis showed a strong positive correlation (*R* = 0.815, *p* < 0.001). The sensitivity and specificity of the infrared photograph analysis for detecting small-angle esotropia were 95.2 and 77.9%, respectively, with a cutoff value of 4.0 PD”	“Selection bias may be present, as this is a retrospective cross-sectional study… Not all children could perform the infrared photograph analysis, as they could not tolerate the few seconds with an occluder placed in front of their eyes… a few children (13.7%) were determined with the Krimsky test, which is subject to measurement error. Software is based on normative ophthalmic biometry. Therefore, the analysis of subjects who have extreme proportions falling out of the normal variation was limited.”
Pundlik et al. (56)	Adult; Non-strabismus participants (*n* = 139), Strabismus (*n* = 100); Age range 20–40 years	Smartphone application capable of assessing strabismus deviation through static photography as well as assessing patient performance of cover-uncover or alternate cover test through video camera recording	Corneal light reflex	Primary and intended horizontal deviation	40 centimeters	APCT, modified Thorington test	“The gaze angles measured by the app closely followed the ground truth (slope = 1.007, R^2 = 0.97, *p* < 0.001), with a root mean squared error (RMSE) of 2.4*Δ*. Phoria measurements with the app were consistent with MT (slope = 0.94, R^2 = 0.97, *p* < 0.001, RMSE = 1.7Δ). Overall, the strabismus measurements with the app were higher than with Synoptophore (slope = 1.15, R^2 = 0.91, *p* < 0.001), but consistent with CTPN (slope = 0.95, R^2 = 0.95, *p* < 0.001). After correction of CTPN values for near fixation, the consistency of the app measurements with CTPN was improved further (slope = 1.01).”	“Use of population average [Hirschberg ratio (HR)] can lead to an error in individual measurements… It is likely that there are age-, sex-, or ethnicity-related differences in HR values… We also did not evaluate the effect of glasses on the accuracy of the app.”
Zheng et al. (113)	Adult and pediatric; Strabismus patients (*n* = 19); Age range unspecified	Remote infrared video camera recording automated cover test	Iris/limbus, pupil	Primary	33 centimeters	“[T]he ground truths of deviations in prism diopters were provided by manually observing and calculating the deviations of eyes for all samples.”	“Experimental results demonstrate that the deviation of strabismus can be well-evaluated by our proposed method. The accuracy was over 91%, in the horizontal direction, with an error of 8 diopters; and it was over 86% in the vertical direction, with an error of 4 diopters.”	“For the acquisition of data, there are obvious changes in the video brightness, due to the cover of the occluder. This might bring a perturbation for the algorithm, especially for the pupil detection. Second… a slight movement of the head that is not detectable to humans will cause a certain deviation in the detection of eye position, thus, reducing the accuracy of the final evaluation.”
Luo et al. (28)	Strabismus patients; *n* = 14; Age range unspecified	“Smartphone app to perform Hirschberg test for measuring manifest and intermittent ocular misalignment”	Corneal light reflex, iris center	Primary	40 centimeters	Prism and alternate cover test	“As the linear regression analysis showed (slope = 1.02, R^2 = 0.94, *p* < 0.001), the app measurements of strabismus angles were consistent with clinical cover test measurements.”	“The app only provides magnitude of the misalignment, rather than any interpretation or diagnosis… Usually for patients with larger eye fissures, i.e., iris area being more revealed, the fitting will be robust and accurate. On the other hand, for patients with smaller eye fissures… the fitting may be prone to inaccuracies. The current version does not provide measurement of vertical misalignment…”
Maio et al. (69)	Non-strabismus participants (*n* = 5), Strabismus patients (*n* = 12); Age range “above 6 years old”	Head-mounted virtual reality headset with infrared video camera	Pupil tracking, eyeball diameter	Primary	“Minions” toy at 6 meters	Prism cover test	“The mean difference between the two techniques and the doctor’s results for all of the patients were all less than 0.7 degrees.”	“In some cases (such as young children), the headset is too big, which produces estimation errors…[Regarding] eyes that were too small or lush eyelashes that covered the pupil, the pupil center could not be accurately tracked.”
Yehezkel et al. (11)	Pediatric; Strabismus patients; *n* = 69; Age range 3–15 years	Remote infrared video camera	Vector between pupil and corneal light reflection	Primary	50 centimeters	APCT and cover-uncover test	“A high correlation was found between the automated and the manual test results (R = 0.9 and *p* < 0.001 for the horizontal deviation, and *R* = 0.91 and *p* < 0.001 for the vertical deviations, with 100% correct identification of the type of deviation). The average automated test duration was 46 s. The Bland–Altman plot, used to compare the 2 measurement methods, showed a mean value of −2.9 prism diopters (PD) and a half-width of the 95% limit of agreement of ±11.4 PD.”	“…This method cannot be applied to patients with a large amplitude of nystagmus - not designed to measure torsion…could not distinguish dissociated vertical deviation (DVD) from vertical hypertropia.”
Zheng et al. (47)	Pediatric; Non-strabismus participants (*n* = 3,021), Strabismus patients (*n* = 2,772)	Static photographs and deep learning algorithm for assessment of misalignment	Region of interest including pupils, iris, conjunctivae, eyelids, and rectangular regions encompassing the eyes.	Primary	Unspecified	APCT and Hirschberg test	“Using 5-fold cross-validation during training, the average areas-under-the-curve of the DL models were approximately 0.99. In the external validation data set, the DL algorithm achieved an AUC of 0.99 with a sensitivity of 94.0% and a specificity of 99.3%. The DL algorithm’s performance (with an accuracy of 0.95) in diagnosing referable horizontal strabismus was better than that of the resident ophthalmologists (with accuracy ranging from 0.81 to 0.85).”	Does not perform quantitative assessment of strabismus; Assessment of strabismus is limited to primary gaze.
Cheng et al. (51)	Pediatric; *n* = 113; Unknown strabismus status; Age range <18 years	Smartphone application capable of assessing strabismus deviation through static photography	Corneal light reflex	Primary	40 centimeters	APCT	“The nurse obtained at least one successful app measurement for 93% of children (125/133). 40 were flagged for PACT, of which 6 were confirmed to have strabismus. Based on the ROC curve, the optimum threshold for the app to detect strabismus was determined to be 3.0△, with the best sensitivity (83.0%), specificity (76.5%).”	Experiment limited to assessment of strabismus in primary gaze.
Yeh et al. (53)	Adult and pediatric; Strabismus patients (*n* = 38); Age range 13 to 65 years	Head-mounted infrared video camera with 3D virtual reality headset	Position of the central pupil in relation to corneal light reflex	Primary	6 meters	APCT	“The angle of ocular deviation measured by the VR-based system and the APCT showed good to excellent correlation [intraclass correlation coefficient, ICC = 0.897 (range: 0.810–0.945)]. The 95% limits of agreement was 11.32 PD.”	“The sample size was relatively small. We did not correct for patient’s refractive error…we only compared the measurement by the VR-based system with that of the APCT, and we did not determine intra-observer and inter-observer data from measurements with the VR-based system. We included all types of strabismus, such as comitant and incomitant strabismus, which might also affect measurements. However, in order to simulate the APCT in a real clinic situation, we only measured the ocular deviation in the primary position.”
Huang et al. (48)	Adult; Non-strabismus participants (*n* = 30), Strabismus patients (*n* = 30); Age range unspecified	Static photograph	Deep learning face detection: pupil position	Primary	Unspecified	Comparison to the results of normal subjects evaluated with the same experimental method	“The average value of the iris positional similarity of normal images was smaller than one of the strabismus images via the method (*p* < 0.001). The sample mean and sample standard deviation of the positional similarity of the normal and strabismus images were 1.073 ± 0.014 and 0.039, as well as 1.924 ± 0.169 and 0.472, respectively.”	“Pupil center…can be unevenly blocked by the lids/lashes, leading to inaccurate center calculation…the localization of the medial and lateral canthus may not be perfectly accurate due to several possible factors (e.g., skin color, illumination, and inapparent facial contour), which has an impact on the measurement of the positional similarity of two eyes.”
Mesquita et al. (59)	Pediatric; Unknown strabismus status (*n* = 204), Strabismus patients (*n* = 22); Age range 5–15 years	Static photograph	Corneal light reflex compared to center of limbus	Primary	40 centimeters	APCT and simple cover test	“Fraction measurements were used with two cutoff points of 6 and 11 prismatic diopters (PD). Results were compared according to their concordances, with a fair Kappa equal to 0.43 [95%CI = (0.38; 0.48)], which was statistically significant (*p* < 0.0001) at the cutoff point of 6 PD and Kappa equal to 0.49 (95% CI = [0.35; 0.61]), which was statistically significant (*p* < 0.042) in the cutoff point of 11 PD.”	“The mhealth application analyzes the images since the eye with a deviation is being observed tangentially to the cell phone’s camera and not at the right angle as interpreted by the ophthalmologist. This results in the contradiction among values found and it will be greater the deviation.”
Garcia et al. (two studies in one paper) (8)	Adult; Unknown strabismus status, *n* = 28; Age range 18–56	Static photograph	Corneal light reflex, limbus for automated reference point	Primary	Near: 33 centimeters, Distance: 4 meters	APCT	“The application obtained a matching rate of 95.14% for the face and eyes. The application yielded a sensitivity of 92.86% for horizontal strabismus at distance and near fixation, however, with low specificity values (7.692, 14.81, and 8%).”	“Since binocular fusion was not disrupted, the application was limited to the measurement of manifest deviations and did not measure latent deviations.”
Garcia et al. (two studies in one paper) (8)	Adult and pediatric; Strabismus patients; *n* = 8; Age range 12–57	Static photograph	Corneal light reflex, limbus for automated reference point	Primary	Near: 33 centimeters, Distance: 4 meters	APCT	“The Bland–Altman plots derived from Study B showed bias values of application measurements between 3.625Δ and 6.125Δ with wide intervals of the limits of agreement. Repeatability of the measurements yielded bias values of −0.625Δ and 2.5Δ for horizontal and vertical strabismus at distance and 4.375Δ and 1.25Δ at near fixation, respectively.”	“Our existing data is skewed toward normal and exotropic subjects, with a lack of subjects exhibiting vertical strabismus – reflecting the population of strabismus patients referred to our clinic.”
Kang et al. (21)	Adult; Strabismus patients; *n* = 2; Age range unspecified	Static photographs and deep learning algorithm for assessment of misalignment	Corneal light reflex, limbus; Ratio of impaired eye movement to normal eye movement	Nine cardinal gaze positions	1 meter	“[T]he areas of the limbi and sclerae for both eyes were manually annotated…for use as the ground truth images.”	“The segmentation models exhibited high performance, with 96.88% dice similarity coefficient for the sclera segmentation and 95.71% DSC for the limbus segmentation.”	“Several assumptions were made based on the limitations of analyzing three-dimensional objects in a 2D environment… (1) Both the limbus and sclera are perfect spheres (2) In adults, the radius of the sclera is 2.5 times longer than that of the limbus (3) The extension line of the corneal light reflex point penetrates the center of the eyeball…It was difficult to identify the location of the limbi in cases of small eyes with little exposure to the limbus area. Potential for the existence of differences according to the age or surgery status of the patient.”
Morrison et al. (41)	Adult; Non-strabismus participants with artificially-induced strabismus; *n* = 10; Age range 26–66 years	Head-mounted infrared video camera	Pupil	Five targets on the tablet (center, vertical and horizontal ± 8.5°).	260 millimeters	APCT	“We found a significant correlation between the reference APCT and the Skew video-oculography (VOG) (Pearson’s R^2 = 0.606, *p* < 0.05). There was a good agreement between the two tests (intraclass correlation coefficient 0.852, 95 CI 0.728–0.917, *p* < 0.001). The overall accuracy of the VOG was estimated at 80.53% with an error rate of 19.46%. There was no significant difference in VOG skew estimations compared with the gold standard except for very small skews.”	“Our current study investigated artificially induced skew on healthy participants. Thus, it has not yet been tested on patients with pathological skews.”
Huang et al. (114)	Adult and pediatric; Non-strabismus participants (*n* = 30), Strabismus patients (*n* = 30); Age range unspecified	Static photograph and meta-learning algorithm for assessment of misalignment	Corneal light reflex	Primary	1 meter	“[Participants] underwent screening tests conducted by a professional ophthalmologist and the screening results were used as the ground truth to evaluate the classification results.”	“The proposed method achieved a classification accuracy of 0.805 with a sensitivity (correct classification of strabismus) of 0.768 and a specificity (correct classification of normal) of 0.842, whereas the classification accuracy of using meta-learning alone was 0.709 with a sensitivity of 0.740 and a specificity of 0.678.”	“[I]mage data only comes from a hospital in Busan; whether the result can be generalized to other regions remains to be verified… Second, images without cornea light reflex are the potential factors that affect classification performance… Third, [there may be] imprecise localization of the medial and lateral canthus due to the factors such as illumination, eyelashes, and inapparent facial contours.”
Luo et al. (76)	Adult and pediatric; Non-strabismus participants; *n* = 207; Age range 5–60 years	Static photographs and convolutional neural network algorithm for assessment of misalignment	Limbus	Nine cardinal gaze positions	100 centimeters	“Manual measurement of the images was conducted…by another experienced ophthalmologist.”	“The intraclass correlation coefficients between manual and automated measurements of six extraocular muscles ranged from 0.802 to 0.848 (*p* < 0.001), and the bias ranged from −0.63 mm to 0.71 mm.”	“Participants with eyelid diseases were excluded from this study, because that abnormality of eyelid function or morphology would cause ocular measurements far from the real values…the effect of eyeball size had not been considered in this study…only participants aged below 60 years were included in the analysis…our deep learning method had not been validated in populations with ocular motility disorders or populations of other ethnicities.”
Rajendran et al. (22)	Adult and pediatric; Strabismus patients; *n* = 39; Age range 3–41 years	Remote video camera performing automated cover-uncover test with infrared glasses	Vector between the corneal light reflex and pupil center	Primary	50 centimeters	APCT	“The prism alternate cover test (PACT) manual measurements and the automated alternate cover test for measuring horizontal deviation, the manual measurement, and the automated eye track system showed a highly positive correlation (*r* = 0.932, *p* < 0.001). The Bland Altman plot analysis shows good agreement between the two measurements, with the mean difference between the two measurements being 1.55 PD, and the 95% limit of agreement was ± 10 PD.”	“[The method] is based on eye movement detection and hence not feasible for patients with paralytic strabismus…measurement is variable in cases with large-amplitude nystagmus…the automated tracker tests deviation only in the primary position of gaze…cannot detect torsion…small sample size…non-inclusion of patients with vertical deviations…measurements were performed for near deviation alone.”
Lou et al. (39)	Adult and pediatric; Strabismus patients; *n* = 72; Age range 4 to 56 years	Static photographs and deep learning algorithm for assessment of misalignment	Height difference between the inferior corneal limbus of both eyes	Adducted position	1 meter	“Manual measurement of IOOA based on the photographs in the contralateral gaze was conducted by an investigator”	“There were significant correlations between automated photographic measurements and clinical gradings (Kendall’s tau: 0.721; 95% confidence interval: 0.652 to 0.779; *p* < 0.001), between automated and manual photographic measurements [intraclass correlation coefficients (ICCs): 0.975; 95% confidence interval: 0.963 to 0.983; *p* < 0.001], and between two-repeated automated photographic measurements (ICCs: 0.998; 95% confidence interval: 0.997 to 0.999; *p* < 0.001).”	“Deviation in the adducted position was not measured by alternate prism cover test…it was difficult to fit the corneal limbus by a full ellipse in a few cases of eyes with little exposure of the corneal area…diversity of sample was limited, because this study included more mild-to-moderate [inferior oblique overactivation] IOOA eyes than severe IOOA eyes…the present study measured IOOA based on two-dimensional photographs, whereas real eyes are three-dimensional.”
Azri et al. (73)	Pediatric; Strabismus patients; *n* = 44; Age range <16 years	Static photograph	Corneal light reflex, kappa angle calculation	Primary gaze	1 meter	APCT	“The correlation between the angle measured by the PCT and the angle measured by Strabocheck® (SK) was strong (R = 0.87). The mean absolute difference in the angle measured by the two methods was Δ = 11.9+/− 9.8 diopters. The Bland–Altman plot shows a 95% interval limit between −30.0 [−34.4; −25.6] and 31.0 [26.7; 35.4] diopters.”	Misalignment assessed only in primary gaze.
Nixon et al. (27)	Adult; Non-strabismus participants (*n* = 7), Strabismus patients (*n* = 19); Age (mean) 58.7 ± 22.4 years	Head-mounted infrared video camera with augmented reality used for an automated alternate cover test	Pupil	Primary	6 meters	APCT	“STARE was able to identify the presence of horizontal strabismus with an area under the curve of 1.00 (100% sensitivity and 100% specificity). The mean difference (bias) {95% CI} was 2.1 {−1.8, 9.9} prism diopters, and the 95% coefficient of repeatability {95% CI} was ±27.9 {14.8, 50.8} prism diopters. The Pearson correlation between APCT and STARE was r24 = 0.62, *p* < 0.001.”	“Headset…too big for use in young children…unable to measure torsion… STARE cannot be used in those with nystagmus…does not differentiate between manifest and latent deviations. Only horizontal deviations were considered in comparisons of size of deviation between APCT and STARE…no intra- or inter-observer data was recorded…ocular deviation was also only measured in primary position with STARE…small sample of patients.”
Gao et al. (55)	Age unspecified; Strabismus status unknown; *n* = 1	Head-mounted infrared video camera with augmented reality and deep learning algorithm for assessment of alignment	Pupil	Six gaze points, unspecified	1 meter	None reported	“The experimental results show that when the distance between the subject and the display is 1 meter, the eye tracking accuracy of the smart glasses can reach 1.0° with an error of no more than ±0.1°.”	Not compared to gold standard evaluation. Largely descriptive paper detailing software design.

### 2.2 Statistical meta-analysis

Sixty-nine studies were identified through database search. Of these, 22 studies either examined Pearson correlation, Kendall’s tau correlation, intraclass correlation (ICC), or kappa statistics between the new techniques for strabismus assessment and the gold standard and/or clinical grading scales (Table 1). Because Kendall’s tau correlation coefficient measures a non-linear association between two quantitative continuous variables, we utilized a technique by Gilpin (29) to convert this correlation into Pearson correlation coefficient. For data characterized by non-continuous variables, Kappa coefficient and ICC were often reported, but they are not on the same scale even though they provide a measure of agreement between two techniques or two raters. In addition, Kappa deals with nominal data while ICC provides a more effective analysis of ordinal and interval data. Nominal data is used to label variables without any quantitative value. It categorizes data by labeling or naming values. The key characteristics of nominal data are: (1) no inherent order- the categories are distinct and separate, with no hierarchy or ranking among them, (2) data consists of mutually exclusive categories- each category is unique, and an item can belong to only one category, (3) uses descriptive names or terms to represent categories, without implying any numerical relationships. Ordinal data is a type of data that classifies variables into categories with a meaningful order or ranking. The key characteristics of ordinal data are: (1) data categories are ranked, having a clear order or hierarchy, such as from high to low, (2) the intervals between categories are not necessarily equal and numerical values can be used as labels, but these values do not represent equal intervals. We therefore reported their results separately from those of Pearson correlation and Kendall’s tau. To obtain a more accurate approximation of confidence intervals around the estimates, all correlations and ICC were transformed using the Fisher’s Z transformation (30):


$$Z=0.5\times \ln\frac{1+r}{1-r},$$

where the standard error is expressed as

$${\mathrm{SE}}_{Z}=\frac{1}{\sqrt{N-3}},$$

and N the sample size of each study.

Additionally, of the 69 studies, 14 reported sensitivity and specificity of the new technique for the diagnosis of strabismus. Of these, three studies also provided a correlation. Because sensitivity and specificity are proportions, a logit transformation was performed before the meta-analysis to ensure approximate normality and variance stabilization.

Heterogeneity was assessed using the Cochran Q test and I-squared (*I*^2^) statistics as described by Higgins and Thompson (31). The *I*^2^ Statistic measures the proportion of total variation in observed effect sizes that is due to variance in true effects rather than random chance. The significance threshold for the Cochran’s Q test is set at alpha = 0.05. Publication bias for meta-analyses in the cases of correlation, sensitivity, and specificity was assessed by the Egger’s tests along with funnel plots. Funnel plots are visual aids for assessing bias or systematic heterogeneity. When random effect models are used, heterogeneity is usually accounted for in the modeling step; thus, the stress in the funnel plot is primarily on the presence or absence of publication bias. A symmetrical inverted funnel shape indicates an unbiased distribution of studies, whereas an asymmetrical shape may indicate selective reporting and/or other systematic biases. The x-axis on the plot represents the observed effect size while the y-axis represents the standard error.

All meta-analyses results were obtained under a random-effect model to allow for heterogeneity in the estimation process. R software version 4.4.2 was used to carry out the meta-analyses. Since the sample sizes available for each meta-analysis is limited, it was not feasible to account for multiple covariates in the estimations of the pooled correlation, intraclass correlation, sensitivity, and specificity. Studies included in the meta-analysis were those that provided, by the parameters described above, objective data from which statistical analysis could be performed. Studies that were not included in the meta-analysis were summarized in Table 1 or narratively evaluated in the discussion.

## 3 Results

### 3.1 Meta-analysis of studies reporting Pearson correlations and Kendall’s tau

**Figure 2:**
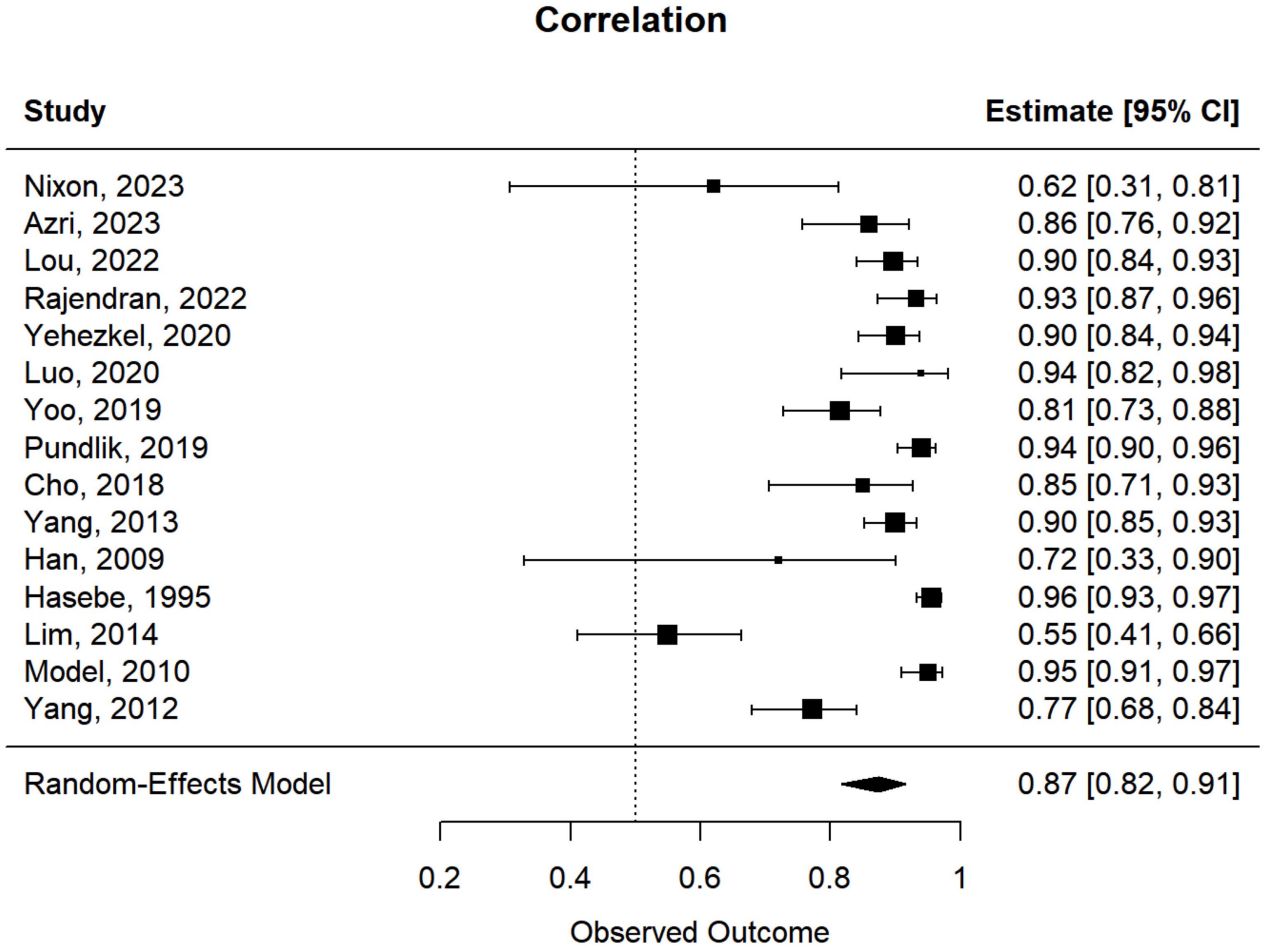
Forest plot of the meta-correlations between new techniques and clinical validation method.

**Figure 3:**
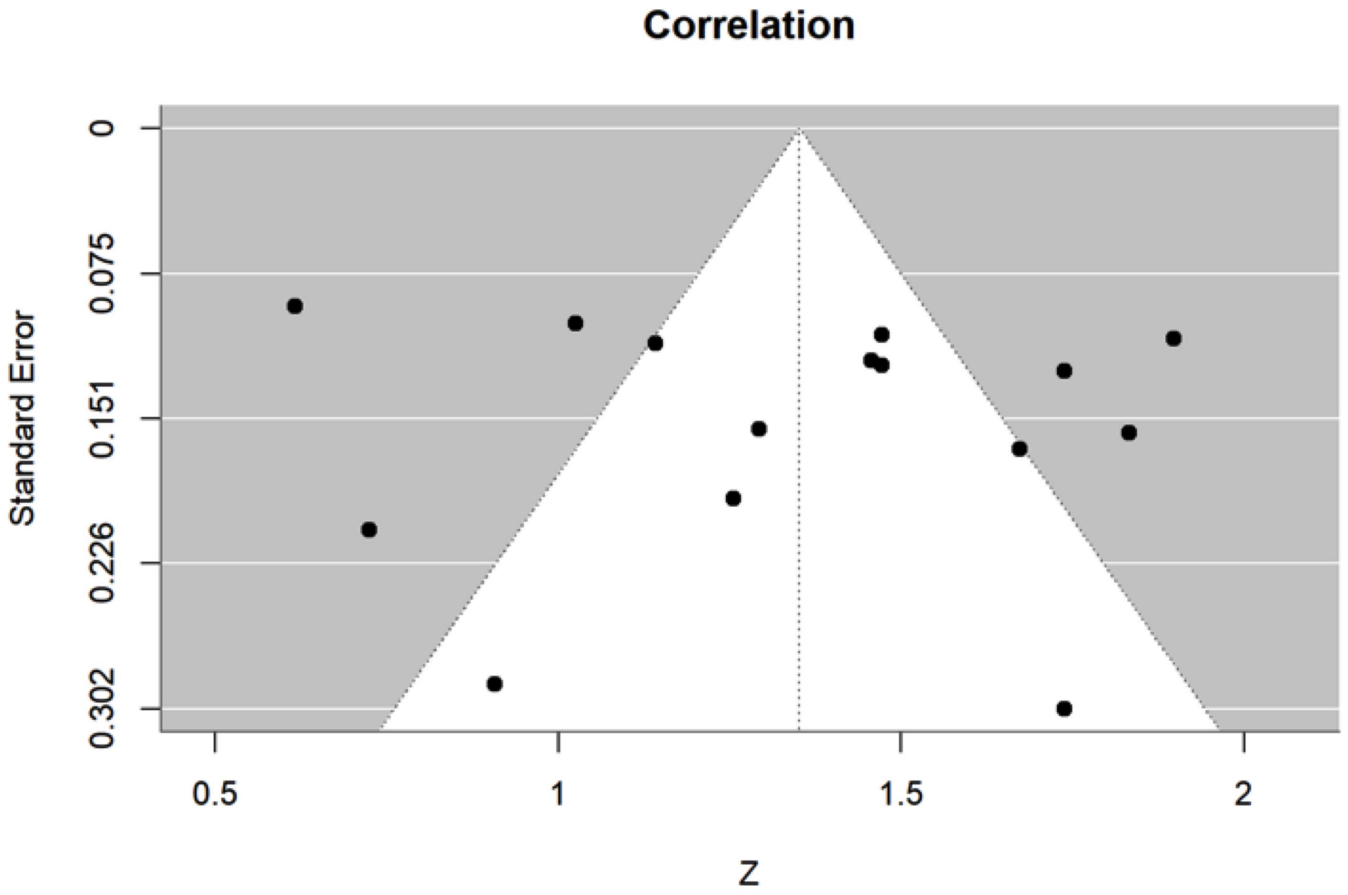
Funnel plot of the meta-correlations between new techniques and clinical validation method. The x-axis represents the effect size of each individual study, which was calculated as Fisher’s z-transformed correlation z$=0.5\ln\frac{1+r}{1-r}$ , and the y-axis represents the corresponding standard error. The white triangular region denotes the expected 95% confidence region in the absence of publication bias.

This meta-analysis included 17 studies out of 69 (24.64%). Their sample sizes ranged from 10 to 158 participants. The average (or median) age of the participants ranged from 2.8 to 58.7 years; and the reported correlations ranged from 0.549 to 0.956 (Table 2). Among the included studies, Nixon et al. (*r* = 0.62), Lim et al. (*r* = 0.549), and Yang et al. (*r* = 0.772) reported the lowest correlation values (27, 32, 33). Specifically, Nixon et al. evaluated an augmented reality headset with eye-tracking in a relatively small sample (*n* = 26), with measurements limited to horizontal deviations in primary gaze only, which may have introduced instability in correlation with APCT (27). Lim et al. used a clinical grading scale rather than APCT as the validation method, potentially introducing subjectivity and lowering the observed correlation (32). While static photograph methods have shown strong correlation with APCT in other studies, Yang et al. included a wide age range (0.5 to 58 years), suggesting possible variability in cooperation or diagnostic visibility across age groups (33). Figure 2 shows the estimates and confidence intervals post meta-analysis. The pooled estimation of the correlation was 0.87 [95% CI: (0.81, 0.91)]. In this analysis, the Cochran’s Q test for heterogeneity had a *p*-value of <0.001 and the *I*^2^ statistic was large (98.34%), suggesting that obtaining the overall estimate via a random effect model would provide an effective analysis. The funnel plot appeared approximately symmetric around the pooled estimate and the Egger’s test was statistically insignificant (*p* = 0.800; see Figure 3), indicating there was no evidence of publication bias. In other words, smaller studies did not consistently report stronger or weaker effects, which suggests that the results were less likely to be distorted by selective publication and supporting the robustness of the pooled findings.

**Table 2:** Characteristics of studies with Correlation/Kappa/ICC.

Year	Country	Age^a^ (Yrs)	New technique	Validation method	Sample Size^b^ (Number of strabismus Subjects)	Correlation (r)/Kappa/ICC
Nixon et al. (27)	United Kingdom	58.7 ± 22.4^1^	Augmented reality headset with integrated eye-tracking	APCT	26 (19)	r = 0.62
Azri et al. (73)	France	7.83 [3.58–17.3]^2^	Static photograph	PCT	44 (0)	r = 0.86
Lou et al. (76)	China	17.6 ± 12.7^1^	Deep learning-based image analysis	Hand measurement by ophthalmologists of same photographs provided to deep learning analysis software	72 (72)	r^c^ = 0.897 ICC = 0.975
Rajendran et al. (22)	India	13.64 ± 9.04^1^	Automated cover-uncover test	APCT	39 (39)	r = 0.932
Tenório et al. (59)	Brazil	[5–15]^3^	Static photograph	APCT	224 (22)	Kappa = 0.43
Yehezkel et al. (11)	Israel	7.17 ± 2.78^1^	Automated cover-uncover test and automated prism cover test	Cover-uncover test and APCT	69 (69)	r = 0.9
Luo et al. (28)	United States	NR^4^	Smartphone app-automated Hirschberg test	PCT, APCT	14 (10)	r = 0.97
Yoo et al. (75)	South Korea	2.8 ± 1.2^1^	Static photographs	APCT	83 (83)	r = 0.815
Pundlik et al. (56)	China	13 [4–63]^2^	Static photographs taken by smartphone app	APCT	66 (66)	r = 0.97
Chopra et al. (57)	United Kingdom	Strabismus: 55 [IQR 33–66.5] Healthy: 50 [41–59]	Binocular OCT	APCT	30 (15)	r = 0.85
Yang et al. (60)	South Korea	7.3^4^	Infrared images obtained using selective wavelength filter	APCT	60 (60)	r = 0.9
Han et al. (71)	United States	29 [19–65]^2^	Automated gaze tracking	APCT	15 (0)	r = 0.849
Hasebe et al. (34)	Japan	17.5 ± 22.9^1^	Video-enhanced Hirschberg test	APCT, PCT	87 (87)	r = 0.956
Yeh et al. (53)	China	39.4 ± 16.0^1^	Eye-tracking virtual reality headset	APCT	38 (38)	ICC = 0.897
Weber et al. (38)	Switzerland	37 [6–18]^5^	Automated Hess screen test using binocular infrared video goggles	Hess screen test	58 (41)	ICC = 0.83
Lim et al. (32)	South Korea	11.2 ± 10.8^1^	Static photograph	Clinical grading scale	120 (120)	r = 0.549
Model and Eizenman (101)	Canada	27 [18–57]^2^	Automated Hirschberg test	Clinical Hirschberg ratio	43 (0)	r = 0.95
Yang et al. (78)	South Korea	6.6 [0.5–58]^2^	Static photograph	APCT	33 (33)	r = 0.772

NR, Not Reported; ICC, Intra-class Correlation; APCT, alternate prism cover test; PCT, prism cover test. ^a^Age is expressed as ^1^mean ± SD (Standard Deviation), ^2^Mean [Min-Max], ^3^[Min-Max], or ^4^Mean, or ^5^Median [Min-Max]. ^b^ Study size represents the number of participants evaluated using both the new technique and the validation method for Correlation, Kappa, or ICC. This number may differ from the total study sample size in the corresponding publication. ^c^ This correlation value was converted from Kendall’s tau (τ = 0.721).

### 3.2 Studies reporting intraclass correlations

**Figure 4:**
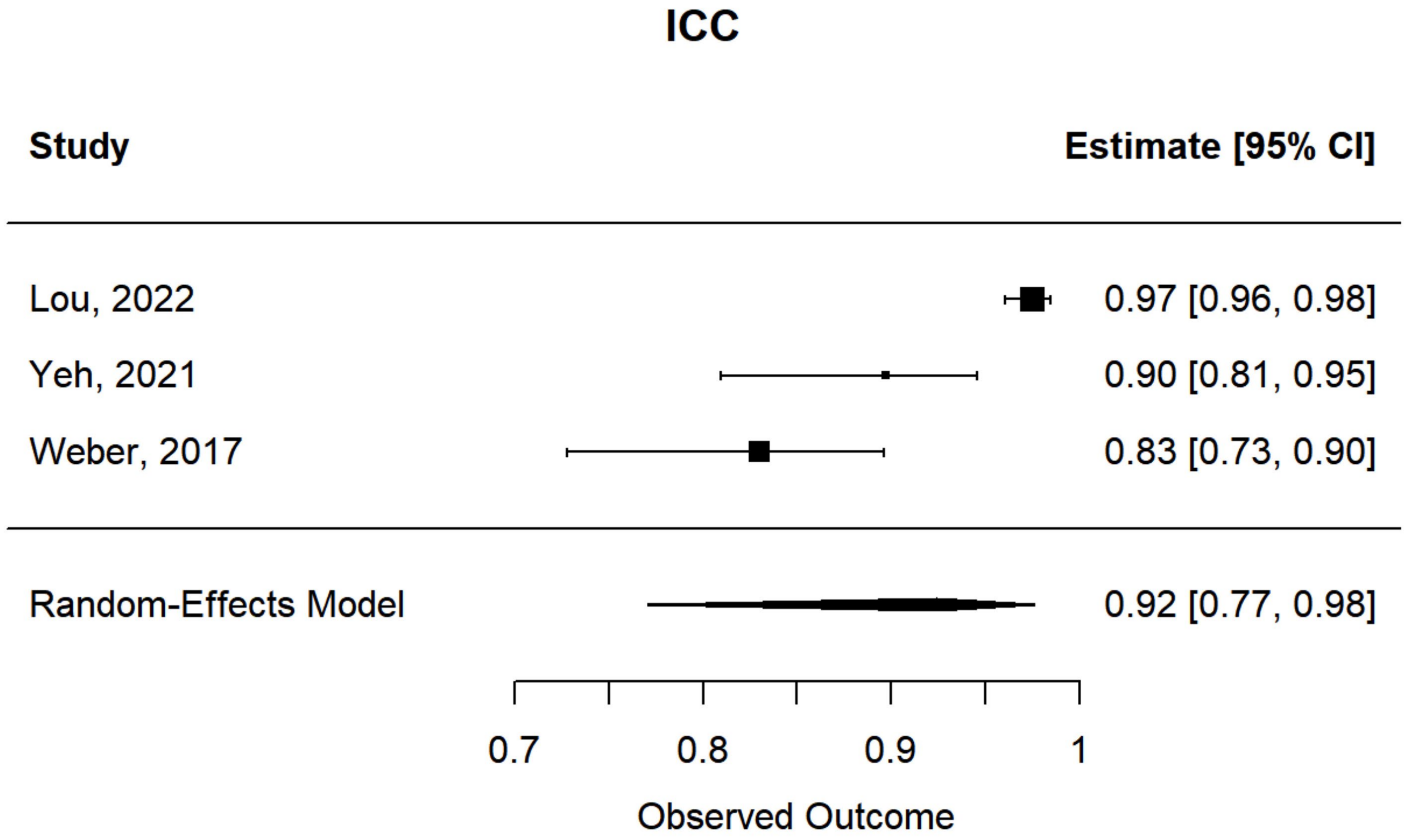
Forest plot of meta-ICCs between new techniques and validation methods.

We proceeded similarly with the studies reporting ICC (3 out of 69, i.e., 4.3%). As seen in Figure 4, the overall estimate of the ICC was quite high, around 0.92 [95% CI (0.77, 0.98)]. This suggests that the new technique and the clinical validation method provided highly consistent measurements in the same individual across studies. In other words, the new technique can reasonably reproduce results from the clinical validation.

### 3.3 Studies reporting sensitivity and specificity

**Figure 5:**
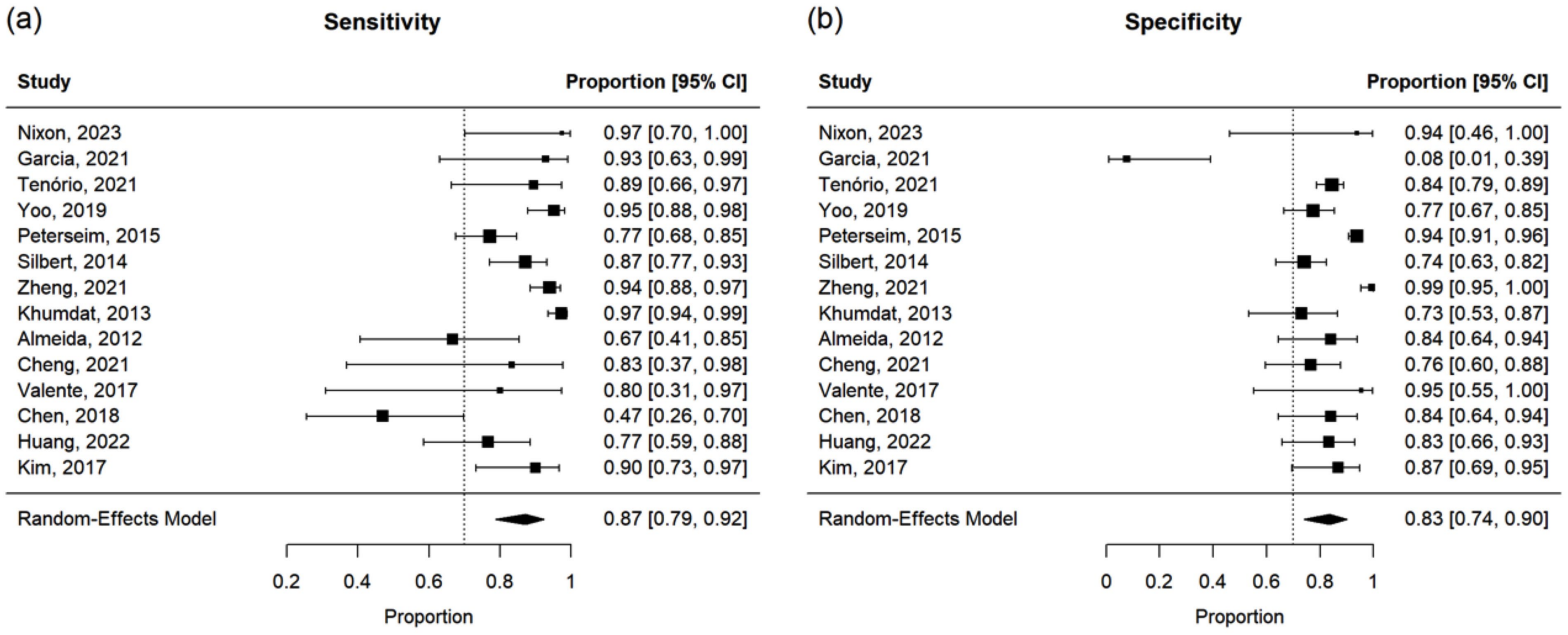
Forest plots of **(a)** sensitivity and **(b)** specificity for all studies in meta-analysis. Notes: An adjustment was applied to account for potential bias in two of the papers that reported a sensitivity or specificity of 100%. Specifically, 0.5 was added to each term for sensitivity calculations when TP (True Positives) or FN (False Negatives) equaled 0, and for specificity calculations when TN (True Negatives) or FP (False Positives) equaled 0.

**Figure 6:**
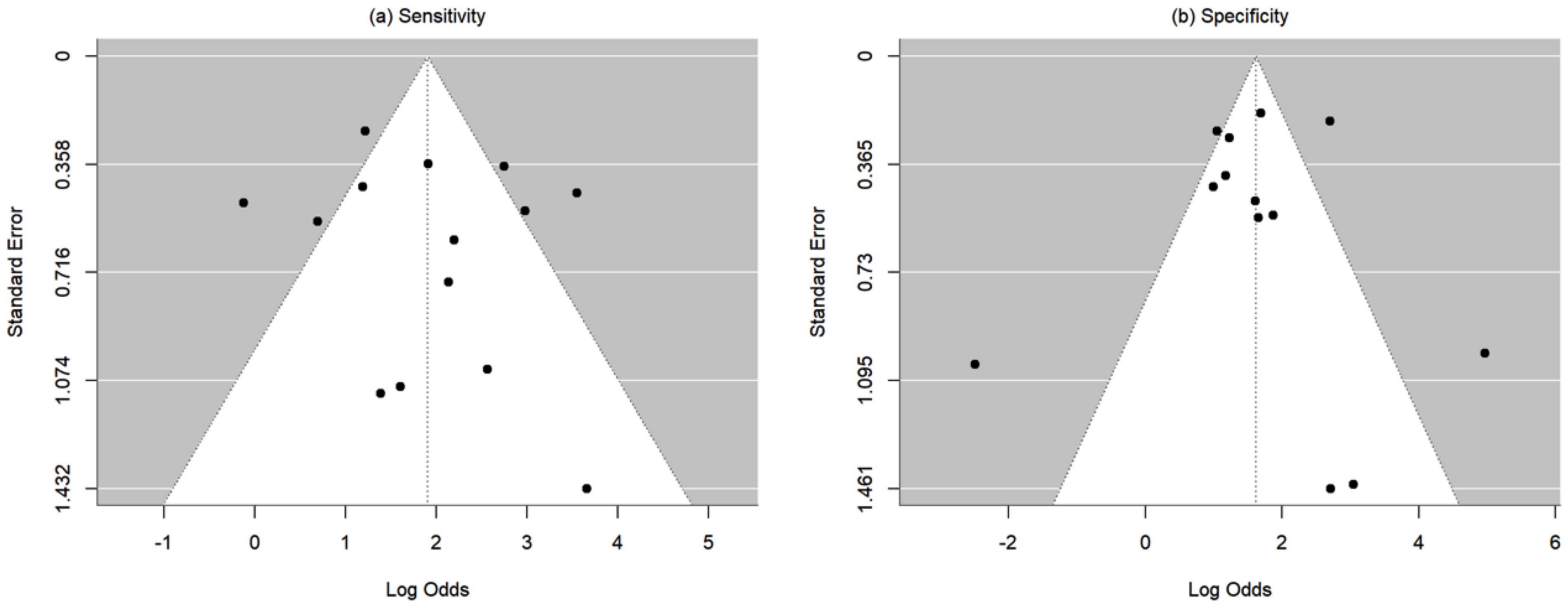
Funnel plots of **(a)** sensitivity and **(b)** specificity. Sensitivity and specificity were transformed to log odds before pooling. The x-axis represents the log-transformed odds in each individual study and the y-axis represents the corresponding standard error. The white triangular region denotes the expected 95% confidence region in the absence of publication bias.

Seventeen out of 69 studies (24.63%) evaluated the diagnostic performance of their new method compared to the validation method. Among these, three studies did not focus on detecting strabismus exclusively. These were not included and the remaining 14 (20.28%) were utilized. These studies were published between 2012 and 2023 (Table 3). The sample sizes ranged from *n =* 15 to *n =* 443. The average (or median) age of the participants ranged from 2.8 to 58.7. The sensitivities and specificities reported in those studies, respectively, ranged from 47.1 to 100%, and from 7.7 to 100%. This wide range likely reflects differences in methodology, technology, and sample size. It is also important to realize that sensitivity and specificity are influenced by the make-up of the patients being tested, in terms of severity of strabismus and could explain differences between studies, besides instrumentation. For example, Garcia et al. (8) reported high sensitivity (92.86%) but extremely low specificity (7.69%) using static photograph method. This estimate was based on only one true negative in a small sample (*n* = 27), making the specificity calculations highly sensitive to misclassification and potentially less reliable. Figure 5 displays the forest plots of the estimates and confidence intervals from the meta-analyses along with the pooled sensitivity and specificity. The sensitivity and specificity of the new diagnostic technique were, respectively, 0.87 [95% CI: (0.79–0.92)] and 0.83 [95% CI: (0.74–0.90)]. Substantial heterogeneity among the included studies was observed as indicated by the *I*^2^ statistic of 77.82% for sensitivity and 85.41% for specificity. In addition, the Cochran Q test revealed heterogeneity in the analyses of both sensitivity and specificity (*p* < 0.001). This validated the use of the random effect model for estimation. Though there were some points falling outside the write triangular region, the funnel plots for sensitivity and specificity are roughly symmetric and suggest a low risk of publication bias (Figure 6). This was further supported by the results from Egger’s test, which showed no evidence of publication bias (*p* = 0.5223 for sensitivity; *p* = 0.8040 for specificity). These results suggested that smaller studies did not appear to systematically report stronger or weaker effects, which strengthens confidence in the robustness of the pooled estimates for sensitivity and specificity.

**Table 3:** Reported values from studies reporting sensitivity and specificity.

Year	Age (Yrs)	New technique	Validation method	TP	TN	FP	FN	Sensitivity	Specificity
Nixon et al. (27)	58.7 ± 22.4^1^	Augmented reality headset with integrated eye-tracking	APCT	19	7	0	0	100.00%	100.00%
Garcia et al. (8)	37.89^4^	Static photograph	APCT	13	1	12	1	92.86%	7.69%
Tenório et al. (59)	[5–15]^3^	Static photograph	APCT	17	173	32	2	89.47%	84.39%
Yoo et al. (75)	2.8 ± 1.2^1^	Static photographs	APCT	79	58	17	4	95.20%	77.90%
Peterseim et al. (108)	6 [0–18]^2^	Static photograph	“Motility evaluation” by ophthalmologist	71	329	22	21	77.17%	93.73%
Silbert and Matta (36)	[0–6]^3^	Static photograph	“Pediatric ophthalmic examination”	61	60	21	9	87.14%	74.07%
Zheng et al. (47)	NR	Deep learning algorithm using primary gaze photographs	APCT	125	143	1	8	93.98%	99.31%
Khumdat et al. (103)	14 ± 3^1^	Static photograph; strabismus detection from corneal light reflex	“Examination” by “three specialists”	175	19	7	5	97.22%	73.08%
Almeida et al. (72)	NR	Static photograph with automated Hirschberg test	Cover test	10	21	4	5	66.67%	84.00%
Cheng et al. (51)	NR	Static photograph through smartphones application of Automated Hirscberg test	APCT	5	26	8	1	83.33%	76.47%
Valente et al. (52)	NR	Automated cover test	Prior “specialist diagnosis”	4	10	0	1	80.00%	100.00%
Chen et al. (110)	[25–63]^3^	Gaze tracking through convolutional neural network	“Diagnosis by ophthalmologist”	8	21	4	9	47.06%	84.00%
Huang et al. (114)	NR	Static photographs recording corneal light reflex	“Screening by ophthalmologist”	23	25	5	7	76.80%	84.20%
Kim et al. (33)	46.75 [5–74]^2^	Combined Manual and Computerized Lancaster red-green test for cyclotorsion	Lancaster red-green test, double Maddox rod test	27	26	4	3	90.00%	86.67%

TP, True Positives; TN, True Negatives; FP, False Positives; FN, False Negatives; NR, Not Reported; APCT, alternate prism cover test; PCT, prism cover test. Age: Expressed as ^1^mean ± SD (Standard Deviation), ^2^Mean [min-max], ^3^[min-max], or ^4^Mean.

## 4 Discussion

### 4.1 Meta-analysis demonstrates a strong correlation between automated strabismus evaluation and expert clinical assessment

Our meta-analysis of 17 studies that qualified for objective pooled analysis showed a pooled estimation of correlation at 0.87 [95% CI: (0.81, 0.91), *p* < 0.001; Figure 3], with reported Pearson correlation of individual studies ranged from 0.549 to 0.956 (Table 2). Additionally, of the 14 studies that reported sensitivity and specificity for analysis of strabismus exclusively (versus the additional detection and analysis of other diagnoses), the sensitivity and specificity of the new diagnostic techniques were 0.87 [95% CI: (0.79–0.92)] and 0.83 [95% CI: (0.74–0.90)], respectively. However, the studies also exhibited notable heterogeneity, with the sensitivities and specificities ranging from 47.1 to 100%, and from 7.7 to 100%, respectively (see Results). Differences between studies may be explained by the composition and severity of the strabismus in the patient populations tested as well as instrumentation and testing factors. These results demonstrate the significant range of accuracy and reliability in automated measurement in previous years and show promise for the development of devices with the capacity to be integrated into clinical use in the future. While detection of strabismus is useful for guiding patient referral, complete and accurate characterization of strabismus through automated means is important for remote triage and the formulation of an appropriate diagnosis and treatment plan.

### 4.2 Advantages and limitations of current devices capable of automated strabismus evaluation

The challenge of designing automated strabismus technology with the capacity for implementation into ophthalmology clinics has spanned over decades (27, 34, 35). The rationale for performing automated strabismus measurement is multi-faceted, including the opportunity to provide an objective, image-based measurement of alignment in place of a subjective, manual evaluation. Additionally, image-based technological algorithms have the potential to provide faster testing times, resulting in a more rapid flow of patients through clinic or triage. Furthermore, an instrument which allows operation by relatively untrained healthcare providers would serve as an avenue of outreach to medically underserved areas and to clinics that lack expertise in the evaluation of strabismus, but where prompt triage is necessary. In remote or understaffed settings, automated strabismus evaluation would ideally perform an assessment providing a complete quantification and characterization of strabismus. Additionally, where applicable, an accurate and comprehensive automated diagnostic workup of strabismus could be integrated into a triage protocol in remote settings that could expedite transfer to a higher level care center for further treatment.

The range of capabilities of instruments designed to evaluate ocular misalignment include screening for unspecified abnormalities in ocular alignment, focused analysis of a known diagnosis, or a more comprehensive analysis of ocular dysmotility with the goal of a diagnosis (32, 36–38). For example, Silbert et al. tested a device capable of detecting amblyopia risk factors, including strabismus, in children with a sensitivity and specificity of 87 and 74%, respectively (36). However, the technology was not designed to quantify the degree of strabismus, and children identified as at-risk would require additional manual testing to confirm a diagnosis (36). In contrast, Lou et al. produced a deep learning-based photographic analysis capable of quantifying binocular misalignment, however the algorithm created is specific to strabismus caused by inferior oblique muscle overaction (39).

Ideally, an objective measurement would provide precise quantification of ocular misalignment to a greater degree than subjective measurement currently obtained via the gold standard alternate prism cover test. Regarding device capabilities, a broad screening for abnormalities and a measurement of targeted conditions can be useful in areas that can refer patients to clinical experts. However, in areas where referral may be delayed or unavailable, it would be necessary for automated analysis to provide a more comprehensive assessment of ocular alignment and motility. To this end, this review discusses major constituents of instruments designed for automated strabismus evaluation. Additionally, this article provides recommendations for designing a comprehensive method of automated strabismus evaluation that is also affordable, accessible, and portable.

### 4.3 Remote versus head-mounted tracking systems

#### 4.3.1 Remote eye-tracking systems

There are reported advantages and disadvantages of remote (the imaging device is not attached to the patient) versus head-mounted eye-tracking systems. For testing small children or infants, a remote eye-tracking device or screen may be preferred (40). Young patients or patients with sensory issues may not tolerate wearing a head-mounted device, and fitting a head-mounted instrument onto a smaller-sized head can be difficult (40). Remote testing may also offer a more convenient option in triage for patients with traumatic head injuries who cannot wear a head-mounted device (41). Another advantage of remote testing is the manual control with which an examiner may create near versus far fixation by physically moving the instrument or target screen in relation to the patient (42). Other arguments in favor of a remote testing model have been made regarding the testing of torsional strabismus, during which head roll independent of the testing camera may provide a more straightforward and accurate measurement of torsion (43).

Conversely, remote devices present several disadvantages compared to head-mounted apparatuses. While a remote device allows physical adjustment of near versus far fixation with potentially less robust technology, this in turn requires a relatively large amount of clinic space to perform accurate distance measurements. Compared to head-mounted systems, the precise position of the head may be uncontrolled when using remote tracking, unless head tracking is implemented along with eye tracking. Change in head position during testing can cause inaccurate quantification of strabismus if it is not accounted for by head tracking in remote video recording systems (21, 44, 45). High accuracy is vital when measuring ocular deviation, as even small changes in position measured in pixels may decrease the validity of results (46). Implementation of head tracking would also enable a gaze position to be changed by just changing the position of the head while the subject fixates on a central target, similar to what is done in clinical assessment with cover testing.

Additionally, inaccurate line-up between the eye and the tracker is a problem encountered in photographic, video, and deep learning models (21, 46, 47). Zheng et al. demonstrated this issue following their analysis of primary gaze photographs analyzed by deep convolutional neural networks (DCNN) that screened for horizontal strabismus (47). In cases that were misclassified by the DCNN, misclassification was attributed to the eyes stationed off-center of the photograph due to head tilt or roll. It is feasible that similar concerns would also apply to slight movements forward or backward, or to any pitch or yaw (47, 48). The use of chin rests to limit movements of the head has been tested to solve this problem (49). However, this additional equipment may decrease the portability of the system (32, 49, 50) and make it less tolerable for testing children. Help may also be enlisted from additional examiners or assistants who monitor the position of the patient’s head (11). A more robust solution would be the use of multiple camera positions so that head and eye position can be rendered in three dimensions (3D) with corresponding software. This would allow assessment of eye and head position in 3D space as a function of gaze position. Additionally, more robust software can implement algorithms to account for slight changes in head position (34, 46, 51). Guyton et al. worked to overcome this challenge by implementing two separate trackers that track the eyes and the movement of the head, separately, and by implementing algorithms to account for the discrepancy during analysis of ocular alignment (44).

Images recorded from remote devices capture a broader picture than that of head-mounted goggles which are limited to the area around the eyes. Image frames of the entire face of the patient as well as the surrounding environment are often recorded and subsequently must be processed for a system to identify the features of the eyes to be tracked. Image processing to eliminate noise while maintaining resolution incurs additional computational cost (20). Additionally, the surrounding testing environment may affect the image of the patient such as lighting, shadowing, or reflections of surrounding objects or sources of light (28, 52).

#### 4.3.2 Head-mounted eye-tracking systems

Compared to remote testing methods, head-mounted virtual reality headsets can block visual input outside of the periocular area and provide control over the testing environment. This advantage eliminates potential interferences such as room lighting, unwanted reflections on the ocular surface, or visual distractions that may draw the patient’s attention away from the test (27, 38). Additionally, any movement of the head will occur in conjunction with the tracking camera, supporting continuous alignment. However, proper fitting of the headset throughout testing is vital, since slight movement of the headset with respect to the eyes during testing will render calibration of gaze position inaccurate (27). Headsets designed for the average adult-sized head may prevent testing of the pediatric population, although there are reports of hardware that can be fitted to the heads of children (38). Additionally, headset-based testing in young pediatric patients may also pose the risk of removal or manipulation of the device by the patient, which can cause interference with data collection or data loss (19). In all cases, it is important to ensure proper fitting for measurement of inter-pupillary distance, as under or over-estimations can affect the measurement of horizontal strabismus (53).

Limitations of head-mounted devices exist regarding the motion of the head. While the headset may prevent the influence of pitch and yaw, a roll of the head may affect measurement by inducing torsion, therefore interfering with evaluation of strabismus involving the fourth cranial nerve (54). To this end, either the patient, a human assistant, or some degree of head constraints would remain responsible for maintaining the upright position (54). Another solution proposed by Nesaratnam et al. is to record the degree of roll if it occurs during testing and to employ software to account for any corresponding torsional component (54).

By design, head-mounted models may more easily test for near deviation, and evaluation of distance deviation would require an artificially induced fixation point (22). Regarding near deviation, Yeh et al. noted an esotropic tendency in their measurements with a virtual reality headset with a fixation point at 75 cm compared to alternate prism cover testing (APCT) (53). While the exact cause is not known, they speculate that a fixation point that is too near, or less than 6 meters, in the virtual reality headset may induce accommodation, convergence, and a corresponding esotropic tendency during testing (53). Other considerations for headsets include ensuring proper fit, as headsets designed for an average-sized head may not fit all patients (38). Furthermore, incorporating correction for refractive error has been a goal and challenge for headset models, as glasses or individual lenses are typically incompatible with a virtual reality goggle design, and not all patients wear contacts (38). Some headsets, such as the virtual reality headset tested by Nixon et al., permitted the wearing of corrective lenses due to its spacious design (27). Weber et al. reports to have overcome the limitations of most cases of refractive error by using a laser target that is theoretically bright enough to be tracked without corrective lenses, although they noted an exclusion of patients with visual acuity less than 20/400 in their study (38). Recently, Gao et al. proposed a model of wearable eye-tracking glasses with settings that accommodate for differences in pupillary distance and for myopia up to −5.00 diopters (55). Focusing on a fixation target without controlling for accommodation may result in accommodative/convergence eye movements that can adversely affect the strabismus measurement (55).

### 4.4 Static photographs versus video-based image recording

#### 4.4.1 Static photograph-based analysis

Data for automated strabismus measurement may be obtained via static photos or by video recordings of eye movements. The benefits of analyzing static photos include the relative ease of use of this method, as photographs with high resolution may be obtained with a commercial-grade, handheld camera or through a smartphone application (8, 28, 48, 51, 56). The use of commercial and handheld devices also offers the benefit of portability (51, 56). Static photos may also provide a rapid mode of testing to quantify specific, known types of strabismus, as demonstrated by Lou et al. in their quantification of the degree of inferior oblique muscle overaction (39). Overall, modern smartphones are equipped with relatively high image resolution, and as shown by Pundlik et al., smartphone cameras may in theory provide a more accurate resolution and measurement of misalignment than gold standard clinical evaluation with prisms and the naked eye (56). These advantages, combined with the small amount of time required to take one or multiple photos, can be useful for young, distractable patients, otherwise non-cooperative patients, or those who cannot tolerate a lengthy clinical exam (20, 37). However, sufficient cooperation with photographs is not guaranteed, as Lim et al. observed during their study, which excluded a reportedly large number of children due to poor cooperation, even with encouragement and the use of toys to promote attention and ocular fixation (32).

Concerns arise when considering the use of static photographs when a comprehensive strabismus evaluation may be needed. For photographs only taken in primary gaze position, the patient is not evaluated for deviations in all gaze positions or for incomitant strabismus (8, 28, 39, 57). In addition, patients with eccentric fixation or an atypical angle-kappa (the angle between the visual axis and the pupillary axis) may give false positive strabismus results (58). Theoretically, static photographs provide data only for end-gaze results, and eye movement cannot be assessed (21, 47). Along these lines, as discussed by Garcia et al., the position of the eyes at the moment of photographic image capture may be slightly different than the end point measured with the APCT, resulting in small yet significant discrepancies (8).

Another commonly documented limitation of evaluation with static images is the inability to assess latent deviations, or phorias (8, 59). Luo et al. developed an application designed to overcome this limitation, and they described software capable of detecting and photographing an eye at the moment an occluder is removed, thus capturing the theoretical degree of phoria (28). Yang et al. approached this challenge by developing an occluder with a selective wavelength filter that blocks the patient’s view of visible light, thus inducing a latent deviation, while permitting the transmission of infrared light used to photograph the eye and measure the degree of phoria (60).

#### 4.4.2 Video-based analysis

Video-based techniques are beneficial in that they allow the assessment of alignment during ocular movement and evaluate movement during occlusion for the quantification of latent strabismus (27, 61). Logistically, video-based mechanisms are better equipped than photographs to assess intermittent strabismus, nystagmus, saccades, and smooth pursuit movements (27, 61). Additionally, video recording allows the examiner to monitor fixation, gaze position, and visual alignment either in retrospection or in real-time via live streaming (11). Video assessment may lessen the burden of work on low-tolerance patients, as recording may allow quicker testing and replaying of the movement of interest instead of the repetitive, time-consuming movements of the prism cover tests (11, 22, 34). To this end, many video-based eye tracking systems report testing times within seconds to minutes (22, 41, 52, 62). As Nixon et al. explained, in addition to offering an objective measurement of gaze deviation, video eye tracking also holds the potential to quantify misalignment to a more precise degree than is currently possible with prisms (27). Mestre et al. echoed this goal of video-based measurement, as previous literature describes the limit of eye movement detection by the unaided human eye as around 2 prism diopters (PD) (61, 63, 64). The combination of video-based eye tracking systems with a head-mounted design, such as the device used by Cantó-Cerdán et al., especially supports precision by reducing inaccuracies caused by head movement relative to the camera (65).

Disadvantages of video-based systems include the need for more robust technology (compared to the technology required to obtain static photographs) which may limit portability, affordability, and operating ability by non-experts in remote clinical settings (52, 66). To counteract this concern, Valente et al. reported a lower-cost design where video results may be analyzed on a remote “workstation computer.” (52) With the consumer demand for virtual reality devices for entertainment and gaming, newer video-based eye tracking devices are now available at much lower cost (52). Regarding measurement accuracy, video technology relies on temporal resolution, which is determined by frame rate, to analyze gaze deviations that can be measured in pixels.(65) Various optimal frame rates for recording eye movement in strabismus analysis have been proposed in the literature, with authors reporting success from rates of 30 Hz - 250 Hz (41, 66, 67). With higher frame rates, the dynamics of eye movements including saccadic velocity and waveform may provide additional diagnostic information allowing one to categorize strabismus into paralytic, restrictive, or neuromuscular junction etiologies (68).

### 4.5 Mechanism of eye tracking

#### 4.5.1 Pupil tracking

Automated strabismus devices use various eye-tracking methods by targeting the pupils, corneal light reflections, corneal limbus, retinal birefringence, red reflexes, or by using optical coherence tomography (OCT) (21, 37, 47, 49, 57, 69). Benefits of pupil tracking include its ease of video-based tracking, however this method assumes that the position of the pupil correlates with gaze direction (69). Additionally, when infrared lighting is used for gaze tracking, the pupil provides a robust contrast from the surrounding iris for threshold-based image segmentation, which is reported to have a low computational cost (36, 38, 69). Considerations for pupil tracking include ensuring the individual determination of interpupillary distance and axial eye length for accurate measurement of deviation (36).

Eyelid blinking may interfere with pupil tracking, since, as Nyström et al. noted, blinking in adults typically occurs at a rate of 20 times per minute with durations of 150–400 milliseconds (67). For accurate measurement, software features capable of removing the effects of the blink on the image frames are needed as is the accurate estimation of the pupil borders even when partially occluded by the eyelid, especially in downward gaze (52, 69). Positioning of cameras from below the visual axis may help reduce the challenge of tracking the pupil in downward gaze. Interference with pupil tracking has also been reported due to the presence of dark eyelashes, mascara, or dark-colored irises that interfere with threshold-based pupil detection, or long eyelashes that cover the area of the pupil on camera (69). Anatomical abnormalities that can interfere with pupil detection and tracking include anisocoria, iris coloboma, extreme axial length, or irregular vertex distance (27, 36, 69). Smaller eyelid fissures or ptosis can also interfere with imaging the full circular pupil shape (21, 36, 69). Seo et al. also noted the potential effects of ambient or test lighting on pupil size during testing as well as the change in pupil size that occurs during the cover-uncover test (62). They recommend dim lighting to promote pupil dilation and lessen the change in size during testing maneuvers (62). However, too large of a pupil will increase the chance of eyelid interference with accurate quantification of the pupil center during tracking. Another confounding variable in accurately tracking the pupil in extreme gazes is the optical effect of the cornea on the true size and location of the pupil center when the camera angle with respect to the eye position becomes significant (70).

#### 4.5.2 Limbus tracking

Limbus tracking has also been explored as an eye-tracking method in both static photographs and video recording (32, 71). Advantages of this strategy over pupil tracking include avoiding the potential for dark irises or eyelashes to interfere with threshold-based detection and the asymmetry imposed by anisocoria or coloboma. However, limbus tracking can also be affected by small eyelid fissures and blinking, or extreme gaze deviations that prevent the tracking of the desired limbal location (32, 69, 71).

#### 4.5.3 Corneal light reflex-based tracking

Other systems utilize a corneal light reflex for eye-tracking purposes, typically in association with an automated Hirschberg examination (49, 72). Azri et al. stressed the importance of measuring the angle kappa, which could skew horizontal strabismus measurements (8, 73, 74). This is reiterated in the literature, as Schaeffel et al. suspected that even small differences in the angle kappa between the two eyes of one subject could affect measurements of alignment (66). Kang et al. exemplified the accuracy with which eye tracking can be accomplished by measuring the difference between the corneal light reflex and the limbus center in photos of the nine cardinal gaze directions (21). The model they described also has the potential to evaluate patients with paralytic strabismus, a known challenge in movement-based eye tracking, as their deep learning model analyzed the difference between the position of the two eyes in patients with fourth nerve and sixth nerve palsies (21).

The use of the corneal light reflex may pose several challenges during strabismus testing (20, 72). Compared to the gold standard APCT, the Hirschberg and Krimsky tests are less accurate (73, 75). They are also more susceptible to visual disturbances which may be unrelated to the cause of ocular misalignment, such as fusional control, which can be affected by patient concentration, alertness, and fatigue (73, 75). For methodology based on the Hirschberg test, Hasebe et al. discussed the importance of determining the unique Hirschberg ratio (HR) for each subject, as individual variability of the HR may cause significant measurement error if a HR based on the population average is used (56, 71). Conversely, Pundlik et al. argued that, especially at lower magnitudes of misalignment less than 15 PD, using the population average had little impact on measurement accuracy (56).

It is important to note that the location of the corneal light reflex may vary during testing in patients with an irregular curvature of the cornea or increased anterior chamber depth (71). For this reason, the corneal light reflex would likely not be an ideal testing method for patients who have undergone refractive surgery, due to disturbances to the corneal surface (66). For the design tested by Schaeffel et al., the authors discussed the challenge of spatial resolution limits imposed by pixel size (66). The need for high-resolution photos to detect the pixel-dependent location of the corneal light reflex may reduce accessibility regarding cost of equipment, however it is possible that modern smartphones possess adequate resolution for this purpose (51, 66). For example, one smartphone application tested by Cheng et al. on schoolchildren utilized a computerized Hirschberg test (51). Other considerations when using the corneal light reflection include the possibility of children closing their eyes due to discomfort with the camera flash as well as secondary reflections from the tear film at the inferior lid margin, which have previously caused errors in corneal reflex detection and analysis (51).

#### 4.5.4 Analysis of retinal birefringence and red reflex

Other instruments designed for automated strabismus evaluation have the ability to screen for, but not quantify, strabismus (37, 40). Even though they are unable to provide a complete diagnostic assessment, these devices may be useful in the evaluation of children who can be referred to an expert clinician for further evaluation (40). Since identification rather than quantification of the angle of deviation is the goal, evaluation of misalignment with retinal birefringence has shown to be a rapid and straightforward strategy, as in the case of the Pediatric Vision Scanner, tested by Jost et al., which detected strabismus and amblyopia in children 2–6 years old, and the Pediatric Vision Screener used by Hunter et al. (35, 37, 50) When the patient focuses their gaze onto a polarized laser light, if the target is centered on the fovea, the returning polarization signal from the foveal Henle fibers provides a characteristic frequency. A change in this expected frequency suggests a lack of central fixation (37, 40).

Similarly, the red reflex, as in the Bruckner test, has been employed as a screening tool for the detection of refractive error, amblyopia, and strabismus (49). This screening method is relatively simple, as the examiner looks for any asymmetry between the red reflexes in both eyes (49). Miller et al. discussed the benefits of this screening method, as the relatively steep angle of the foveal pit may induce a difference in the red reflex for fixation deviations as small as 1 degree, which corresponds to about 2 prism diopters (PD) (49). Drawbacks of this method may arise due to an age-related decrease in reflectivity of the internal limiting membrane, leading to additional scattering of light and possible confounding of observed reflex asymmetry (49). Additionally, a difference in angle kappa between the right and left eyes may give a false positive result of strabismus. Luo et al. noted that while these tests may be especially useful for screening schoolchildren in non-clinical settings, the cost of the instruments has likely been a roadblock preventing their widespread implementation (28).

#### 4.5.5 OCT-based measurement of motility

A less commonly studied mechanism for automated strabismus evaluation is binocular OCT. In the system tested by Chopra et al., the corneal vertex reflection was used as a mark of the central image, while a line was drawn to connect the posterior margins of the pupil in both eyes (57). These lines were compared between both eyes, and the angle of difference between the two was denoted as the angle of deviation (57). Benefits of the OCT design include more rapid testing than the APCT, as well as the ability to produce objective, quantitative measurements of misalignment (57). Additionally, focusing of the eyes on different target points allows strabismus analysis in all nine gaze positions (57).

Using a system involving OCT may necessitate training to perform accurate and reproducible OCT imaging, however Chopra et al. described an automated system that they stated does not require specialized training (57). The cost of the system could rank OCT as a less affordable option of automated measurement for low-resource communities, compared to systems which rely on a simple handheld camera, a smartphone, or a video recorder coupled to a commercial-grade computer, for example (51, 54, 56). Furthermore, analysis via OCT is by nature based on static images, and as discussed by Chopra et al., this can exclude the identification of intermittent tropias and phorias (57). All subjects tested with their design had constant strabismus. They suggested future development of a video-based OCT, which could potentially overcome these limitations (57). As with other forms of strabismus analysis, consideration of refractive error is important since uncorrected error can affect the degree of deviation. The authors also proposed that the additional measurement of axial length and visual axis in future versions of OCT-based designs could increase the accuracy of the measurement (57). The time and fixation required for OCT acquisition may not be feasible for use in children.

#### 4.5.6 Deep learning algorithms

With the rise of artificial intelligence, the creation of deep learning (DL) techniques has become a significant step in the development of automated strabismus evaluation. DL techniques have been used in various experiments to identify a variety of eye diseases, including pediatric cataracts and retinopathy of prematurity, as well as strabismus, with promising results (39, 47). While deep learning algorithms can provide rapid and accurate assessment, the set-up of these algorithms requires baseline input and can necessitate a significant time requirement from specialists. For example, the study on DL assessment of ocular movements by Lou et al. was made possible by the work of two ophthalmologists who outlined the corneal limbus and eyelid margins on the facial images of 1862 volunteers (3,724 eyes), which served as the basis for the training of the eye segmentation network (76). Also, in order to effectively train DL networks, large numbers of images showing both normal and pathological conditions are often required. For instance, in their initial stage of development, Lou et al. used 30,000 facial images for facial segmentation training (76). Zheng et al. also utilized 7,026 images of normal and strabismus patients for the creation of their DL algorithm for the detection of horizontal strabismus in primary gaze photographs (47). Fortunately, with the modern existence of online patient databases from hospitals and clinics worldwide, obtaining relatively large numbers such as these is often feasible. Attention should also be directed to the potential effects of ethnicity or the region of the world from which such images are collected, as noted by Zheng et al., as the exclusive use of images from a common ethnicity may affect the generalizability of certain algorithms (47).

#### 4.5.7 Assessment of torsional strabismus

There is a gap in the literature regarding accurate and feasible automated testing of ocular torsion (33, 43). Torsional strabismus causes ocular misalignment as well as deficiency or difficulty in determining the position of the head relative to the surrounding environment (77). Torsional strabismus can also interfere with an examiner’s ability to assess the functionality of the vestibular system in the context of head rotations, particularly during rolls (77). During torsional measurements using search coils and contact lenses, slippage is a known problem that can affect the accuracy of measurements (77). Alternatively, during more recent image or video-based torsional assessment, interference by noise and artifact has been observed (77). Kim et al. point out that among the various methods for assessing ocular torsion, including the Lancaster red–green test (LRGT), double Maddox-rod test (DMRT), unmounted double Bagolini lenses, synoptophore, and torsionometer, the DMRT and LRGT are two of the most common (33). However, the DMRT presents some limitations, such as limiting the amount of light entering the eye during testing, which may alter some accommodative actions of the eyes relative to their accommodations in daily life (33). Additionally, torsional measurements with the LRGT may not detect minute amounts of torsion, and they can be limited by large amounts of horizontal and vertical strabismus (33). To overcome this, Kim et al. proposed and tested a method that combined elements from both common tests, such as utilizing red-green glasses to subjectively align parallel lines for the determination of cyclotorsion in each eye (33). Additionally, in cases where the eyelids may occlude part of the iris, which is used to detect and track anatomic landmarks during video-based measurement of torsion, Otero-Millan et al. proposed an algorithm capable of recognizing parts of the iris which are either visible to the camera or covered (77). This allowed their system to accurately estimate the position of targeted regions of the iris to assess torsion (77). Separately, Bos et al. addressed the issue of tests that use minute anatomic landmarks within the iris to calculate the pupil center, which can be susceptible to error due to flux in position from the sphincter and dilator muscles (43). They proposed a model that identifies diametrically positioned landmarks within the iris, which provided an averaged measurement of the pupil center and reduced measurement error (43). While this literature review did not conduct an exhaustive search of papers discussing the measurement of ocular torsion specifically, the diagnosis of this form of strabismus is an integral aspect of comprehensive strabismus assessment, and future developments of portable and accessible automated torsional assessment will benefit from the insights gained from previous research, as well as a future in-depth review.

### 4.6 Fixation target design and testing strategy

#### 4.6.1 Simple point design versus image as a fixation point

When designing a virtual reality protocol that uses a fixation target to direct the eye movement of the patient, the design of the target should be considered. Targets for visual tracking in the literature vary from a simple shape measuring a few millimeters in diameter (41) to an image of a cartoon character, as used by Miao et al. (69) Targets that are too small may be difficult for some patients to see and track. Moreover, Nixon et al. explained that a single fixation target on a uniform, non-stimulating background may affect the perceived fixation distance and result in undesired accommodation, convergence, and false measurements of esotropia during testing (27). However, larger targets or targets with multiple points of interest for fixation, such as an image of a recognizable object, may allow minute movements of gaze within the bounds of the target region that could cause lapses in fixation on the very center of the target (69). Novel fixation targets that initially are large when first seen and then rapidly shrink in real time to a smaller target may provide one approach. More investigation is needed to determine an optimal testing target and structured background that promotes steady fixation and fusion in binocular subjects while minimizing induced convergence or phoria.

#### 4.6.2 Testing strategy

In order to validate an automated test for quantifying strabismus, it would seem advantageous to first try and replicate what is done during clinical measurement with prisms using single and cross-cover testing. For virtual head-mounted devices that have a separate visual input for each eye, it is relatively easy to produce a binocularly fused image on a structured background and then virtually “occlude” one eye or the other by eliminating the fixation target in one eye, while still recording the position of both eyes simultaneously. This would also facilitate a built in calibration done during the actual test, assuming that the subject is fixating on the target seen. Since, for example, the field of view of some virtual reality head mounted devices is on the order of 20–30 degrees from fixation in the horizontal and vertical planes, then this would constitute the limit of gaze induced strabismus (61). The other limitation is the quantity of an extreme gaze that can be accurately tracked by the software from the video image (70). For remote devices, where the video cameras are removed from the subject, gaze extremes can be greater by positioning the head in different positions while the subject maintains fixation on a central target, similar to what is done with clinical measurements (42). This would require simultaneous head tracking along with eye tracking to determine gaze position accurately.

#### 4.6.3 2D imaging versus 3D model of the eye

Image-based analysis of ocular misalignment must account for the fact that image and video representation of the eyes are most often 2-dimensional (2D), while the structure of the eyes is 3-deminsional (3D) (21, 27, 60). Considering that the 2D movement of the eyes in the nine cardinal gaze positions actually represents the eyes’ rotation around an axis, at least some degree of measurement error is likely to be inherent with a 2D analysis (21). Yang et al. proposed a software capable of quantifying the angle of strabismus based on a computerized, 3D model of the eye built from 2D photographs (60, 78). Developments such as these point to the benefits of a real-time display of a 3D model of the eye, which could provide test administrators with real-time analysis of eye movements as well as the ability to monitor technological function during the test, rather than retrospective analysis of results alone. This function could provide both important 3D visualization of the eye for diagnostic purposes as well as decrease testing time by allowing premature termination and re-starting of the test as needed in the case of user or system error (21, 60). Real-time 3D modeling of the eye and strabismus measurement can be accomplished if there are at least 2 video camera vantage points during the testing and recording. Therefore, one important design consideration for future instrumentation would be to incorporate multiple, synchronized miniature video cameras to render the eye features in 3D.

#### 4.6.4 Testing duration and ease of use

A software design that is easy to use is an important component of accessibility and portability. One goal of automated strabismus evaluation is a shorter testing duration compared to the gold standard clinical evaluation (1, 11). A review of the literature reveals widespread success toward this goal, as the majority of proposed testing designs are capable of completing testing and producing results within seconds to minutes (60, 62, 69, 75). For example, Nixon et al. presented an automated strabismus screening test requiring only 60 s, and Morrison et al. described a more comprehensive automated alternate cover test which lasts 15 min, which is comparable to a typical clinical testing time with prisms (27, 41). Additionally, Miao et al. developed a virtual reality-based exam which lasts between 1–2 min, among other authors with similarly rapid testing times (60, 62, 69, 75).

Ideally, non-expert or even non-clinical personnel would be able to operate a device that performs automated strabismus evaluation in the setting of a remote clinical or non-clinical setting where prompt triage is necessary. In published studies where instruments are operated by experienced clinical or research staff, the question remains regarding the ability of lay individuals or ancillary personnel to operate the test (37, 51). Silbert et al. aimed to overcome this limitation with the Spot Vision Screener, which reportedly uses visual cues to aid inexperienced operators in obtaining a focused image (36). Rajendran et al. also developed a model that produced results that “do not require expert evaluation or interpretation.” (22) Miao et al. also described the development of a graphical user interface that can apparently be operated by personnel who are inexperienced in the realm of strabismus evaluation (69). Applications available for use on smartphones also support accessibility and portability, as seen in the EyeTurn app by Pundlik et al. and the mobile health application (mhealth) by Mesquita et al. (56, 59) Unfortunately, as Huang et al. acknowledged, even simple technological designs that require nothing more than for patients to take photographs of themselves outside of a clinical setting may still have limitations regarding accessibility, especially in regions of the world that lack access to the internet or even the most common forms of technology (48).

#### 4.6.5 Analysis and exam report

Just as important as simplicity and ease of testing is the data analysis and clinical report needed to convey test results. One starting point is to design a clinical test report that is similar to what orthoptists, pediatric ophthalmologists, and neuro-ophthalmologists now use to record ocular motility and strabismus measurements in the electronic medical record. Then, additional ancillary information and graphics can be added to further render the report intuitive and easy to interpret. Use of captured video frames in gaze positions incorporated into that patient’s strabismus measurements in prism diopters would be one approach.

#### 4.6.6 Cost

On review of current literature, a topic that is seldom discussed in detail is the cost of hardware and software capable of automated strabismus evaluation and the commercial availability of such instruments. Some clinics and institutions may be able to afford the infrared camera and filters used by Yoo et al. or the liquid crystal shutter glasses designed by Seo et al. (62, 75) For others, a smartphone application or a software compatible with a digital camera and workstation computer as described by Valente et al. would be more cost-effective (52, 56). Furthermore, authors such as Chopra et al., Azri et al., and Nixon et al., among others, utilized open-source software as the basis of their models, which promotes public accessibility since open-source technology is typically lower-cost than commercial software (27, 57, 67, 73). The variety of innovation described in the literature can be beneficial in that different institutions and communities can perform individual cost–benefit analyses for the most effective use of their resources. Commercialization of high-quality head-mounted virtual reality headsets with video-based eye tracking for entertainment and gaming may reduce the cost of such hardware. Currently, there is a great need for more sophisticated software development for testing of strabismus and eye movements, accurate analysis of eye position from video, and optimal report generation.

## 5 Future directions

The field of automated evaluation of ocular movement disorders is rapidly expanding. For example, in recent years, the development of new remote devices capable of 3D eye tracking, including the surrounding structures of the eye such as the eyelids, facial expression, and pupil, has become an endeavor for commercial companies interested in providing accurate gaze tracking services in various environments (79). Other commercial companies have chosen to focus on enhancing the evaluation and diagnosis of neurological and neuro-ophthalmologic disorders using virtual reality-based headsets (80). These devices have the potential to use their video recording data to provide an optimal analysis of eye position, diagnose and quantify conditions such as strabismus, and develop clinical reports that are intuitive for most clinicians (81).

Another limitation of many automated strabismus devices is the inability to evaluate saccadic movements. Conjugate saccadic eye movements are a necessary part of changing gaze direction, and studies have shown significant impairment and disconjugate function of the yolk muscles during saccades in patients with strabismus (82, 83). Saccade evaluation can be a useful tool for assessing dysfunction of extraocular muscles, as in dysfunctional coordination of yolk muscle pairs, or of neural pathways. For example, the optokinetic reflex requires both smooth pursuit and saccadic eye movements, and abnormalities in this reflex can help localize dysfunction among the visual striate cortex, medial superior temporal cortex, and pretectal nuclei, or other structures involved in this pathway (84). Similarly, saccadic abnormalities can assist in diagnosing common neurological diseases, such as progressive supranuclear palsy (PSP), which often involves decreased velocity of vertical saccades, or Parkinson’s disease, which often displays hypometric volitional saccades (84). Multiple studies in the literature have measured normative values for saccadic velocity, and data suggests that there may be a wide range of normative velocities that can be influenced by factors such as age or even time of day (85–87). Many prior studies use a relatively small number of participants (87–89). More recently, Song et al. used eye-tracking technology to evaluate saccadic movements in patients with concussions (90), and Hmimdi et al. studied the use of artificial intelligence in the development of robust, next-generation protocols capable of evaluating and characterizing saccadic movements within a diagnostic context (91). While a focused review of literature pertaining to saccadic analysis is beyond the scope of this review, it is important to note that, as in the case of automated strabismus evaluation, the validation of devices capable of saccadic assessment compared to clinical evaluation is critical for their implementation into clinical use.

A device that could measure maximum velocity for a given amplitude of horizontal and vertical saccades could measure a larger number of normal subjects to characterize normative saccadic velocities. Furthermore, this device would ideally evaluate patients with horizontal and vertical saccadic abnormalities with the primary outcome measure being maximum saccadic velocity for a given amplitude. Measurement of abnormal saccades could then be compared to normative data to assist in the diagnosis of neural pathway disease and to identify the etiology of strabismus, such as whether it arises from restrictive (e.g., thyroid eye disease), paralytic (cranial nerve palsy), supranuclear (e.g., intranuclear ophthalmoplegia or skew deviation), or neuromuscular (e.g., myasthenia gravis) processes.

## 6 Conclusion

This review of the literature provides an opportunity to examine the features of automated strabismus technology that promote accurate and rapid data acquisition, accessibility of testing at non-expert clinics, and cost-effective production. Recommendations on the advantages and disadvantages of prominent design characteristics are summarized in Table 4. Overall, the development of devices capable of automated strabismus evaluation must consider a wide range of design principles that support clinical implementation, with an emphasis the following:

- Accurate tracking of eye position and movement in all gaze directions
- Usability by adult and pediatric patients
- Portability and accessibility.

**Table 4:** Advantages and disadvantages of design features common among devices capable of automated strabismus evaluation.

Design	Design Subcategory	Advantages	Disadvantages
Camera position	Remote	May be more tolerable for small children, patients with disabilities, or recent head trauma	Decreased precision of head position relative to testing camera Influence of pitch or yaw may require additional equipment in form of head restraints
	Head-mounted	Can control visual input outside of periocular area Decreases influence of pitch and yaw on measurement	Headset may fit adults but not small children Roll of the head can still induce ocular torsion Distance vision testing requires artificially induced fixation point Correction for refractive error should be built in
Image type	Static photo(s)	High-resolution photos in nine gaze positions may be obtained with accessible, commercial-grade handheld camera or smartphone Portable and rapid data collection	Unable to assess eye movement Images may not reflect end-point misalignment
	Video	Assesses alignment during ocular movement Lessens burden of work on low-tolerance patients compared to repetitive cover testing: ocular movements able to be viewed multiple times retrospectively	Requires more robust technology Variations in frame rates may affect measurement accuracy Limits assessment of paralytic strabismus when algorithms are based on movement patterns
Anatomic landmark for eye tracking	Pupil	Assumed landmark of center of the eyeball Robust pigment contrast in binary threshold-based imaging	Blinking, small eyelid fissures, dark irises or dark eyelashes may interfere with tracking Pupil size may change during cover-uncover testing Should measure interpupillary distance and axial length
	Limbus	May avoid interference of ptosis, dark eyelashes, dark irises	Tracking affected by small eyelid fissures, blinking, extremes of gaze position
	Corneal light reflex	Useful in tracking misalignment due to paralytic strabismus	Angle kappa must be calculated for each eye
	Retinal birefringence	Relatively rapid identification of misalignment and determination if referral is needed	Incapable of quantifying degree of misalignment
	Corneal vertex reflection (OCT-based)	OCT-based photographs allow for more rapid testing than APCT Strabismus analysis can be performed for all nine cardinal gaze positions	More extensive training may be required for personnel to operate OCT Static OCT images provide limited movement analysis compared to video assessment
Target design	Point	Promotes targeted fixation during testing	Smaller targets may be difficult for patients to track With uniform background, may affect perceived fixation distance and induce undesired accommodation May be impractical for patients with macular disease affecting central vision
	Image	May promote fixation for patients with central vision disturbances Recognizable images may hold interest and fixation of young children	Large targets may provide multiple points of fixation, resulting in movement of gaze within the target region and less accurate eye tracking
Data display	2D	Commercially available technology offers 2D imaging	Inherent image-based measurement error due to incomplete representation of 3D structure of the eye
	3D	More accurate representation of visual and rotational axis of the eye	Typically, greater cost associated with high level image processing and 3D model creation from 2D data acquisition
Testing duration	Short (<15 min)	Rapid results when prompt triage is necessary More tolerable for individuals with disabilities or for young children More efficient clinic flow, more patients able to be seen during triage	Potential for less detailed testing (i.e., one static photograph versus video-based recording of eye movement in all nine gaze directions)
	Long (>15 min)	Potential for more detailed testing (longer testing inherent in video recording versus static photograph of primary gaze, for example)	Delayed results when prompt triage is needed (i.e., cases of trauma) Less tolerable for young children or patients with disabilities
Device modality	Virtual reality headset	Adjustable perceived virtual target fixation distance for near and distance testing Adjustable screen-based fixation targets that may help maintain the attention of pediatric patients	Potentially higher cost, limiting widespread accessibility Designing virtual reality-based headsets that fit both pediatric and adult populations presents a challenge
	Smartphone app	Widely accessible to the public Portability Potentially low-cost	Lack of guidance and standardization regarding use and testing interference (lighting, distance of phone from face, etc.) outside of a clinical setting
	Artificial intelligence software (deep learning algorithm)	Rapid assessment based on training that can involve data input by specialists	Large amount of data input required to construct accurate deep learning algorithm Data from certain geographical regions and not others may affect generalizability of assessment capabilities
User population	Professionals	More detailed and accurate testing with the potential for more specific diagnoses	Operation limited to trained professionals Decreased access to testing for populations with limited resources
	Non-professionals	Greater operating and testing accessibility in regions lacking expert providers	Less detailed testing and less accurate diagnoses
Clinical comparison	Alternate prism cover testing	Gold standard Comparison of de-identified automated measurements to de-identified clinical measurements by orthoptist or ophthalmologist	Time required for gold standard testing Lack of access to trained experts in many regions May not be tolerable for young children
	Hirschberg test, Bruckner method, other measurement of deviation, etc.	Less time required for clinical testing May be more tolerable for young children	Less accurate than gold-standard cover testing with prisms

This table provides suggestions for use in guiding future development of automated strabismus evaluation technology.

In summary, a review of the literature reveals that multiple testing designs provide a range of advantages and disadvantages. Head-mounted designs may be less tolerable for young children or impractical for evaluation of ocular misalignment following head trauma, although they allow for control of visual input (27, 38). In patients who can tolerate head-mounted devices, the stable positioning relative to the camera is advantageous for accuracy and reproducibility of data acquisition (27), provided the device does not shift and change position relative to the head during testing. A remote device capable of compensating for any mispositioning of the head relative to the camera could provide a useful combination of accuracy and tolerability for patients of all ages and also provides the possibility of 3D imaging with multiple camera vantage points (79). This type of device would show promise for the increased accuracy and accessibility that would promote integration into clinical use.

While static photographs allow rapid data collection or measurement of previously diagnosed misalignment, video-based tracking provides the substantially increased benefit of evaluating ocular alignment during movement in all nine cardinal gaze directions (8, 61). Multiple anatomic landmarks exist that can provide accurate eye tracking, and all are subject to interference by surrounding structures of the eye or extremes of gaze position. However, the pupil, considered the representative center of the eyeball, provides a unique signal of gaze direction and contrast to other structures of the eye for threshold-based imaging (69). For these reasons, video-based software that utilizes pupil tracking technology may provide an especially robust pathway toward the detailed strabismus evaluation that is necessary for clinical use. Regarding the design of the visual target, while image-based targets such as the “Minion” cartoon used by Miao et al. may assist in holding the attention of small children, a large target provides multiple points of fixation within a target range (69). A target design of a simple point comprised of limited pixels may provide increased fixation stability and therefore a more accurate measurement of gaze position as a function of target location (41). Although the eye and its rotation along the visual axis occurs in 3D, most instruments accessible for commercial use operate with 2D data acquisition. This results in inherent error while constructing a 3D model of the eye for quantification of position around the visual axis (21). However, 3D models of eye movement constructed from 2D photographs or video can be useful for real-time or retrospective evaluation, and measurements of dimensions of the eye such as interpupillary distance and axial length can increase the accuracy of the 3D model (78).

Decreased testing time decreases workload and fatigue in examiners as well as patients, prompt evaluation during triage, and efficient clinic flow promoting the assessment of more patients in less time. Ease of use for testing equipment is important so that instruments operable by non-professionals can be used in regions where clinical resources and training are scarce (56). To increase widespread access to automated testing devices, the burden of cost should be kept as low as possible without sacrificing testing quality or diagnostic capabilities.

Since the gold standard of strabismus evaluation comprises alternate cover testing with prisms, the ideal validation method for devices capable of automated strabismus measurement would include diagnosis by clinician experts based on orthoptist or ophthalmologist measurements of de-identified patient subjects (8). These results could then be compared to de-identified and randomized results from automated measurements. In summary, this review provides a quantitative meta-analysis and qualitative assessment of previous reports of automated strabismus evaluation published in the literature and provides multi-faceted considerations for future designs of advanced technology capable of automated strabismus evaluation (Table 4).
